# Genome-scale metabolic model led engineering of *Nothapodytes nimmoniana* plant cells for high camptothecin production

**DOI:** 10.3389/fpls.2023.1207218

**Published:** 2023-08-02

**Authors:** Sarayu Murali, Maziya Ibrahim, Hemalatha Rajendran, Shagun Shagun, Shyam Kumar Masakapalli, Karthik Raman, Smita Srivastava

**Affiliations:** ^1^ Department of Biotechnology, Bhupat and Jyoti Mehta School of Biosciences, Indian Institute of Technology Madras, Chennai, India; ^2^ Initiative for Biological Systems Engineering, Indian Institute of Technology Madras, Chennai, India; ^3^ Robert Bosch Centre for Data Science and Artificial Intelligence, Indian Institute of Technology Madras, Chennai, India; ^4^ School of Biosciences and Bioengineering, Indian Institute of Technology Mandi, Mandi, Himachal Pradesh, India

**Keywords:** camptothecin yield, metabolic engineering, genome-scale metabolic model, enzyme overexpression, *Agrobacterium tumefaciens* transformation, strictosidine synthase

## Abstract

Camptothecin (CPT) is a vital monoterpene indole alkaloid used in anti-cancer therapeutics. It is primarily derived from *Camptotheca acuminata* and *Nothapodytes nimmoniana* plants that are indigenous to Southeast Asia. Plants have intricate metabolic networks and use them to produce secondary metabolites such as CPT, which is a prerequisite for rational metabolic engineering design to optimize their production. By reconstructing metabolic models, we can predict plant metabolic behavior, facilitating the selection of suitable approaches and saving time, cost, and energy, over traditional hit and trial experimental approaches. In this study, we reconstructed a genome-scale metabolic model for *N. nimmoniana* (NothaGEM *i*SM1809) and curated it using experimentally obtained biochemical data. We also used *in silico* tools to identify and rank suitable enzyme targets for overexpression and knockout to maximize camptothecin production. The predicted over-expression targets encompass enzymes involved in the camptothecin biosynthesis pathway, including strictosidine synthase and geraniol 10-hydroxylase, as well as targets related to plant metabolism, such as amino acid biosynthesis and the tricarboxylic acid cycle. The top-ranked knockout targets included reactions responsible for the formation of folates and serine, as well as the conversion of acetyl CoA and oxaloacetate to malate and citrate. One of the top-ranked overexpression targets, strictosidine synthase, was chosen to generate metabolically engineered cell lines of *N. nimmoniana* using *Agrobacterium tumefaciens-*mediated transformation. The transformed cell line showed a 5-fold increase in camptothecin production, with a yield of up to 5 µg g^−1^.

## 1 Introduction

According to the World Health Organization, cancer is the leading cause of death worldwide, accounting for nearly 10 million cases in 2020.With the rapid increase in cancer incidence each day, the demand for enhanced production of anti-cancer drugs has been a compelling need of the hour. Camptothecin (CPT) is a potent topoisomerase I inhibitor found in plants, mainly *Camptotheca acuminata* (Wall et al., 1966) and *Nothapodytes nimmoniana*, formerly known as *Nothapodytes foetida* (Wight) Sleumer and *Mappia foetida* Meirs, respectively (Nagaraju Shivaprakash et al., 2014). However, climate change accompanied by excessive deforestation for CPT extraction has rendered these plant species in the endangered species category (Isah and Mujib, 2015). To achieve sustainable production of CPT, rational metabolic engineering strategies can be effective for predicting plant metabolic behavior.

Genome-scale metabolic models (GEMs) capture the known metabolism of a given cell within a mathematical framework (Thiele and Palsson, 2010). A GEM describes the whole set of stoichiometric, mass-balanced metabolic reactions in an organism, as well as the associations between genes, proteins, and metabolic reactions, framed based on genome annotation data (Gu et al., 2019). GEMs can be converted into mathematical formats that are amenable to constraint-based modeling. Constraint-based modeling, an evolving modeling approach, offers a powerful tool for systematizing existing biochemical, genetic, and genomic knowledge into a mathematical framework (Bordbar et al., 2014), enabling the analysis of the metabolic capabilities of various organisms.

GEMs have been studied in some plants including *Arabidopsis thaliana* (de Poolman et al., 2009; Oliveira Dal’Molin et al., 2010), tomato (Colombié et al., 2015; Yuan et al., 2016), and rice (Poolman et al., 2013). While most models describe plant growth and primary metabolism, few models elucidate their secondary metabolism. Compartmentalization and prediction of reactions in secondary metabolism have been reported in Arabidopsis (Mintz-Oron et al., 2012). We previously enhanced alpha-tocopherol production by approximately 10-fold in *Helianthus annuus* metabolism by adapting the available *A. thaliana* genome-scale metabolic model (Srinivasan et al., 2019). Model-driven metabolic engineering approaches have the advantage of exploring a larger variety of targets than those that are immediately adjacent to the metabolite(s) of interest.

Several strategies to enhance the production of alkaloids, such as CPT, have been reported (Murali et al., 2021). Some of them include bioprocess optimization strategies, such as elicitation, optimization of media components, and metabolic engineering strategies, such as overexpression of rate-controlling enzymes in the biosynthetic pathway. In *N. nimmoniana*, overexpression of secologanin synthase, an enzyme in the CPT biosynthesis pathway, has been reported recently to increase CPT yield by nearly 2-2.8 fold (Rather et al., 2020).

In this study, we used a rational metabolic engineering approach to enhance CPT production in *N. nimmoniana.* To this end, we reconstructed the GEM of a closely related species, *C. acuminata*. We assembled this GEM, which we named NothaGEM, by integrating the *N. nimmoniana-*specific biochemical data. Furthermore, *in silico* approaches have been used to identify and rank enzyme targets for overexpression and knockout to improve CPT production. We chose one of the top-ranked predicted targets for overexpression, strictosidine synthase (STR), to generate metabolically engineered *N. nimmoniana* cell lines for the amplified production of CPT.

## 2 Materials and methods

### 2.1 Reconstruction of *N. nimmoniana* metabolic network at genome-scale

The objective of this study was to develop high-CPT cell lines of *N. nimmoniana* using rational metabolic modeling. The first step involved reconstruction of a genome-scale metabolic model for CPT production. Owing to the unavailability of the *N. nimmoniana* whole-genome sequence, the whole genome-sequence of *C. acuminata*, another CPT-producing plant native to China and Eastern Asia, was chosen for this study (Zhao et al., 2017). A draft metabolic model was generated using the whole-genome sequence of *C. acuminata* (Zhao et al., 2017) in ModelSEED, a resource for the reconstruction, comparison, and analysis of metabolic models (Henry et al., 2010). From the whole-genome sequence, the *C. acuminata* protein FASTA sequence was procured and uploaded to https://modelseed.org/plant using the “Upload Plants FASTA” option (Seaver et al., 2018). The COBRA toolbox 3.0 (Schellenberger et al., 2011) for MATLAB (version R2019b, MathWorks Inc.) was used for constraint-based modeling and analysis of the metabolic model.

**Figure 1:**
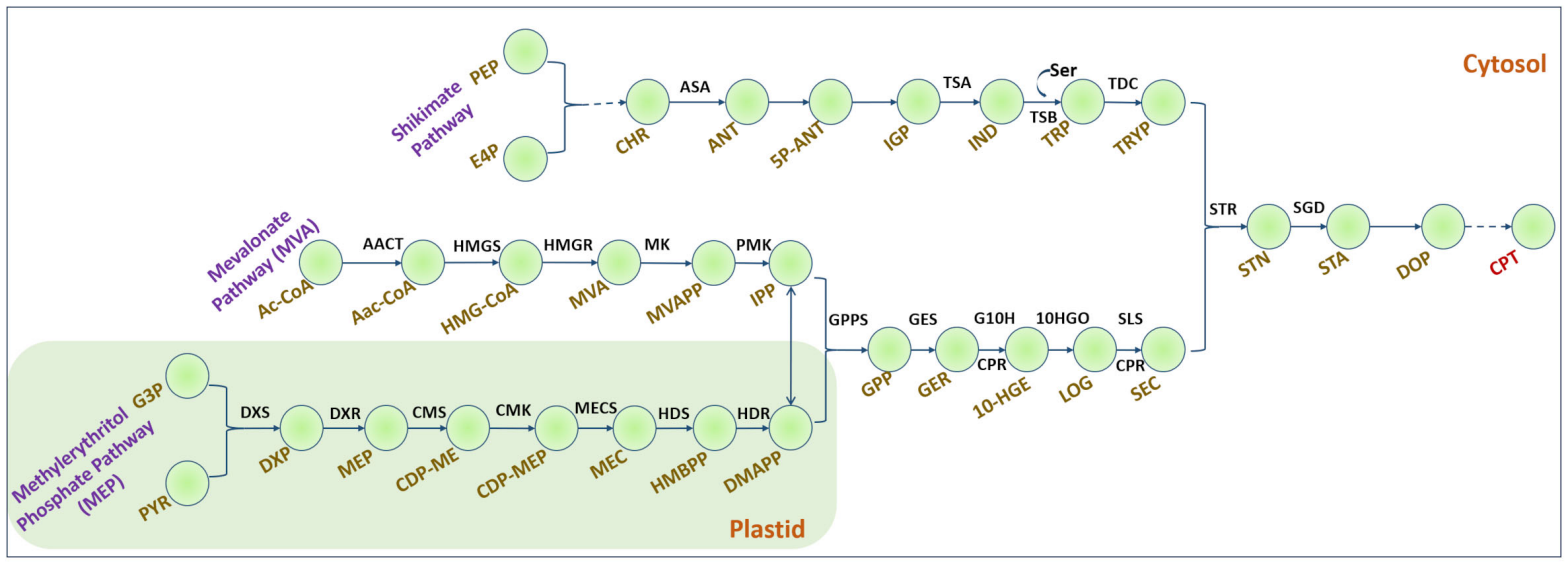
Camptothecin biosynthesis in plants. Dashed lines indicate multiple steps. Shikimate pathway: PEP, Phosphoenol pyruvate; E4P, Erythrose-4-phosphate; CHR, Chorismate; ASA, Anthranilate synthase; ANT, Anthranilate; 5P-ANT, 5-Phosphoribosyl anthranilate; IGP, Indole glycerol phosphate; TSA, α-subunit of tryptophan synthase; IND, Indole; Ser, Serine; TSB, β-subunit of tryptophan synthase; TDC, Tryptophan decarboxylase; TRYP, Tryptamine. Mevalonate pathway: Ac-CoA, Acetyl Coenzyme A; AACT, acetyl CoA: acetyl CoA C-acetyltransferase; AaC-CoA, Acetoacetyl CoA; HMGS, 3-hydroxy-3-methylglutaryl-CoA synthase; HMGR, 3-hydroxy-3-methylglutaryl-CoA reductase; MK, mevalonate kinase; PMK, phosphomevalonate kinase. Methylerythritol phosphate pathway: G3P, glyceraldehyde-3-phosphate; DXS, 1-deoxy-D-xylulose-5-phosphate synthase; DXP, 1-deoxy-D-xylulose-5-phosphate; DXR, DXP reductoisomerase; MEP, 2-C-methyl erythritol-4-phosphate; CMS, 4-(cytidine 5-diphospho)-2-C-methylerythritol synthase; CMK, 4-(cytidine 5-diphospho)-2-C-methylerythritolkinase; MECS, 2-C-methylerythritol-2,4-cyclodiphosphate synthase; HDS, hydroxymethylbutenyl 4-diphosphate synthase; HDR, hydroxymethylbutenyl 4-diphosphate reductase; HMBPP, hydroxy methyl butenyl diphosphate; IPP, Isopentenyl diphosphate; DMAPP, dimethylallyl diphosphate; GPP, geranyl diphosphate; GPPS, GPP synthase; GES, geraniol synthase; GER, Geraniol; G10H, geraniol 10-hydroxylase; CPR, NADPH, cytochrome P450 reductase; 10-HGE, 10-hydroxy geraniol; 10-HGO, 10-hydroxy-geraniol oxidoreductase; LOG, Loganin; SLS, Secologanin synthase; SEC, Secologanin; STR, Strictosidine synthase; STN, Strictosidine; SGD, Strictosidine beta-glucosidase; STA, Strictosamide; DOP, Deoxypumiloside; CPT, Camptothecin.

To reconstruct a metabolic network for CPT biosynthesis (**Figure 1**) organism-specific biochemical data from literature and different databases such as Plant Metabolic Networks (PMN) (Schläpfer et al., 2017) and Kyoto Encyclopedia of Genes and Genomes (KEGG) were collected. In addition, a transcriptome analysis of *N. nimmoniana* revealed 13 genes associated with CPT biosynthesis (Manjunatha et al., 2016). A basic local alignment search tool (BLASTP) analysis (Altschul et al., 1990) performed between the two plants, *N. nimmoniana* and *C. acuminata*, demonstrated similarity between them owing to homology in the camptothecin-producing genes (**Dataset S1 Figure 1**).

Extensive manual curation was performed following standard procedures (Thiele and Palsson, 2010), including gap filling, maintenance of reaction directionality, uniformity in the nomenclature of identifiers, updating metabolite and reaction details (including some biosynthesis reactions of CPT in accordance with the PMN database), adding missing formulae and charges to the metabolites, fixing reaction balances, reaction duplicates, and stoichiometries. We also identified metabolic dead-ends and blocked reactions in the model. Many such reactions are observed in the stroma and cytosolic compartments. Some of these reactions were removed using genomic and experimental evidence (**Dataset S1**). A test suite to assess the quality of the GEM, Metabolic model testing (MEMOTE) was used to evaluate the quality of the final model, NothaGEM (Lieven et al., 2020).

During model reconstruction, a biomass formation reaction was generated, which was validated experimentally. To formulate the *N. nimmoniana-*specific biomass objective function (BOF) (Feist and Palsson, 2010) and determine the biomass precursors, cell suspension cultures of *N. nimmoniana* were lyophilized and analyzed. Cell biomass samples (in replicates, n = 4) were used for metabolite analysis using gas chromatography–mass spectrometry (GC–MS) facility (Agilent Technologies, USA). Biomass precursors are divided into protein, DNA, RNA, carbohydrates, lipids, cell wall components, inorganic ions, and metabolites. The biomass precursors and their coefficients are listed in **Dataset S1**. Growth kinetics experiments were performed to determine the growth of *N. nimmoniana* cells and estimate the specific growth rate. Residual sucrose was estimated using HPLC (Agilent Technologies, USA) with an RID detector to determine the amount of substrate utilized. Total protein content was estimated using the detergent-compatible (DC) assay method, where bovine serum albumin (BSA) was used as a standard. Total lipid content was estimated using the Bligh and Dyer method (Bligh and Dyer, 1959). Using the manufacturer’s protocol, total nucleotides (DNA and RNA) were extracted using Genetix’s DNASure Plant Mini kit and Qiagen RNeasy Plant Mini kit. They were quantified using a NanoDrop spectrophotometer (Thermo Fisher Scientific, USA). Ammonia and nitrate contents were calculated using spectrophotometric assays—Berthelot’s reaction (Solorzano, 1969) and salicylic acid method (Cataldo et al., 1975), where ammonium sulfate and potassium nitrate were used as standards, respectively. Elemental ions such as Potassium, Calcium, Magnesium, Sodium, and Ferrous ion contents were estimated using inductively coupled plasma optical emission spectrometry (ICP-OES, Perkin Elmer Optima 5300 DV).

Flux Balance Analysis (FBA) (Varma and Palsson, 1994; Raman and Chandra, 2009; Orth et al., 2010) is used to calculate the flow of metabolites through a metabolic network and enables us to predict the organism’s growth rate and the rate of production of biotechnologically important metabolites. In our model, FBA was used to analyze NothaGEM by constraining the exchange reactions in the model with experimental values of the substrate and metabolite uptake rates. FBA was performed with the objective function of maximizing cell biomass and the model-predicted specific growth rate was estimated.

**Figure 2:**
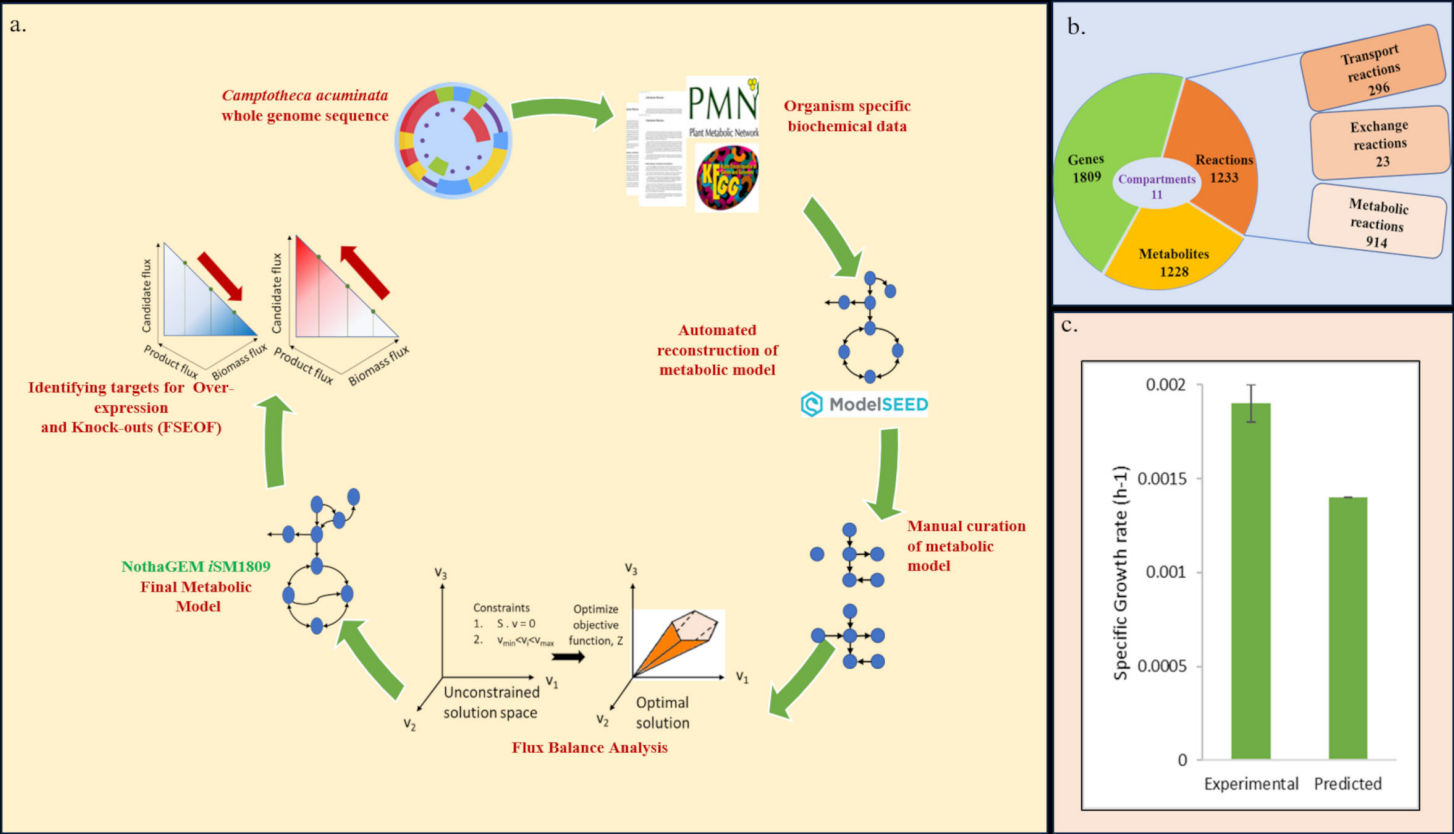
**(A)** Schematic representation of the reconstruction of NothaGEM *i*SM1809; **(B)** Characteristics of NothaGEM *i*SM1809; **(C)** Comparison between the experimental and model-predicted specific growth rates.

Flux scanning-based enforced objective flux (FSEOF) was used to identify targets for overexpression/knockouts and rank them for higher camptothecin productivity (Choi et al., 2010). FSEOF scans for those reaction targets with monotonically increasing fluxes when camptothecin formation is enforced in steps while maximizing growth as the objective function. Biomass was used as the objective function to ensure no significant reduction while enhancing CPT yield. Fluxes of reactions that increase with increasing CPT formation fluxes are ideal targets for overexpression, whereas reactions with decreasing fluxes and a decrease in CPT formation fluxes are ideal targets for knockouts. The potential enzyme targets of overexpression and knockouts were ranked based on a score, ‘*f*,’ a phenotypic fraction weighing the reaction fluxes under the overexpressed and wildtype conditions (Boghigian et al., 2012; Raajaraam and Raman, 2021). The overall steps involved in the identification of overexpression and knockout targets are summarized in **Dataset S1 Figure 2**, and a schematic representation of the computational model reconstruction is shown in **Figure 2**.

### 2.2 Development of metabolically engineered cell lines of *N. nimmoniana*

*N. nimmoniana* plants were procured from https://nurserylive.com/products/mappia-foetida-kalagaura-narkya-plant (Order No. #BB3H055346). The *in vitro* shoots were initiated and maintained on Murashige and Skoog medium (HiMedia, Mumbai) supplemented with 3% wv^−1^ sucrose and 0.5% wv^−1^ clerigar (HiMedia, Mumbai) at an initial pH of 5.8 with a 16/8 h light/dark cycle (Murashige and Skoog, 1962). From these shoots, wild-type callus cultures were initiated and maintained in MS medium, 3% wv^−1^ sucrose, 2 mg L^−1^ 2,4-dichlorophenoxyacetic acid, and 0.1 mg L^−1^ Thidiazuron. A combination of 2,4-Dichlorophenoxyacetic acid (2,4-D) and Thidiazuron (TDZ) was found to be the best combination for induction and growth of callus in *N. nimmoniana* among various combinations of auxin and cytokinin tested. Hence, the same medium composition was used for maintenance, and no further treatment was performed as a part of this study. These cultures were used to generate new shoots and maintained by subculturing every four weeks.

#### 2.2.1 Developing transformed cell lines of *N. nimmoniana*

To integrate STR into the *N. nimmoniana* genome, *A. thaliana* strictosidine synthase (*At-STR*) cDNA (GenBank accession no. AY062976.1) was obtained from the *Arabidopsis* Research Center (ABRC, Ohio). *A. thaliana* STR was selected to prevent any potential co-suppression with the native STR found in *N. nimmoniana* (Matzke and Matzke, 1995). Additionally, strictosidine synthase and similar proteins have been reported in *A. thaliana* as possible contributors to plant defense mechanisms (Kibble et al., 2010).

**Figure 3:**
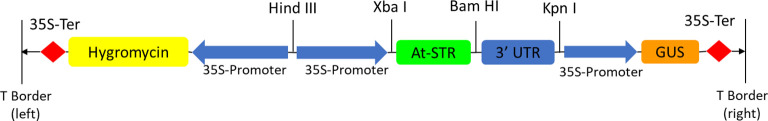
T-DNA region of pCAMBIA 1301 harboring At-STR—an illustration of the overexpression of At-STR in pCAMBIA1301 for heterologous expression in *N. nimmoniana* plant cells *via Agrobacterium*-mediated transformation.

The polymerase chain reaction (PCR) technique was used to clone the At-STR using forward and reverse primers specific to the At-STR designed in the OligoAnalyzer 3.1 tool (Integrated DNA Technologies Inc., USA) mentioned in **Dataset S1 Table 3**. The At-STR was flanked by the double-enhancer version of the CAMV35S promoter and 3’-UTR. The PCR product amplified using the primers was ligated into the region between the unique enzyme restriction sites of XbaI and BamHI in the multiple cloning site (MCS) of the binary vector pCAMBIA 1301, as reported by Srinivasan et al. (2019). pCAMBIA 1301 contains a plant selection marker for the hygromycin (antibiotic) and beta-glucuronidase (GUS) reporter systems (**Figure 3**). The ligated gene, with a size of 1,039 bp in the plasmid, was subcloned into electrocompetent TOP 10 *E. coli* cells and confirmed by sequencing before further use.

*Agrobacterium tumefaciens* LBA4404 was transformed with the binary vector pCAMBIA 1301 containing the insert by electroporation (Bio-Rad Gene Pulser Xcell Electroporation system) at 200 Ω and 2 kV. The transformed *A. tumefaciens* LBA4404 cells with pCAMBIA 1301 were grown in 50 mL yeast extract peptone (YEP) broth (Himedia, Mumbai) with 50 mg L^−1^ rifampicin and 100 mg L^−1^ kanamycin for 24 h in 250 mL Erlenmeyer flasks in an incubator shaker at 120 rpm and 28°C until the OD_600_ reached 0.4. The cells were centrifuged for 10 min at 10,000 rpm and re-suspended in 10 mL fresh MS medium adjusted to a pH of 5.8 to transform *N. nimmoniana* plant cells. *In vitro* leaves and petioles from *N. nimmoniana* shoots were pierced with a sterile needle and suspended in an *A. tumefaciens* suspension in MS medium for 15 min. The co-cultured explants were then blot-dried using sterile filter paper and incubated at 23°C for 48 h in the dark on solidified MS medium (0.5% w v^−1^ clerigar) with 100 μM acetosyringone. These explants were subsequently transferred to Petri dishes containing MS medium supplemented with 3% wv^−1^ sucrose, 0.1 mg L^−1^ thidiazuron (TDZ), and 2 mg L^−1^ 2,4-Dichlorophenoxyacetic acid (2,4-D) for callus induction. Hygromycin 25 mg L^−1^ was used to select transformed plant cells, and 200 mg L^−1^ cefotaxime was used as the antibiotic. Cefotaxime concentration was subsequently reduced to zero in future subcultures after verifying the absence of bacterial growth. Induced calli were subcultured and maintained every two weeks on freshly prepared media. Transformed and untransformed cell lines were subcultured monthly and used for subsequent analysis.

#### 2.2.2 Verification of transgene expression

Genomic DNA of the transformed and wild-type *N. nimmoniana* cell lines was extracted according to the manufacturer’s protocol (Genetix Biotech Asia Pvt. Ltd.). The metabolically engineered cell lines of *N. nimmoniana* harboring At-STRs were verified by PCR amplification using forward and reverse primers specific to At-STRs mentioned in **Dataset S1 Table 3**. Primers were designed using OligoDT Analyzer 3.1 (Integrated DNA Technologies Inc., USA). The PCR reaction mixture included 100 ng of template DNA from the transformed callus, 0.5 µM of each forward and reverse primer, and Phusion High-Fidelity DNA Polymerase (New England Biolabs, USA) made up to a final reaction volume of 25 µL with MilliQ water (Millipore, USA). PCR was performed under the following conditions: initial denaturation at 95°C for 5 min, 25 cycles of amplification comprising (i) denaturation at 95°C for 30 s, (ii) annealing of primers to template DNA at 55 °C for 30 s, and (iii) extension of primers at 72°C for 1 min, followed by a final extension at 72°C for 5 min, in a Veriti^®^ thermal cycler (Thermo Fisher Scientific, USA). The amplified products were analyzed by agarose gel electrophoresis using a 1 Kb DNA ladder (Themo Fisher Scientific, USA) on a 0.8% wv^−1^ agarose gel using Tris-acetate-EDTA as a running buffer and visualized under UV light in Bio-Rad Gel Doc XR+ (Bio-Rad, USA).

Semi-quantitative PCR and quantitative real-time PCR (qRT-PCR) were performed out to verify transgene expression. For this experiment, 100 mg fresh weight of transformed and untransformed calli was used to extract total RNA using the RNeasy Plant Mini Kit (Qiagen, USA), according to the manufacturer’s protocol. The absorbance was estimated using a Nanodrop spectrophotometer (Thermo Fisher Scientific, USA). Approximately 500 ng of RNA were used to synthesize cDNA using the PrimeScript RT reagent kit (TaKaRa Bio, USA). The cDNA was amplified using At-STR gene-specific forward and reverse primers mentioned in **Dataset S1 Table 3**, with *N. nimmoniana* beta-actin as the internal control (Rather et al., 2018). Primers were designed using PrimerQuest™ Tool (Integrated DNA Technologies Inc., USA). The cDNA templates were amplified using semi-quantitative PCR (Veriti Thermal Cycler, Thermo Fisher Scientific, USA) and quantitative real-time PCR (qTOWER, Analytik Jena AG, Germany). The qRT-PCR reaction mixture included 100 ng of template cDNA from the transformed callus, 0.4 µM of each forward and reverse primer, and TB Green Premix Ex Taq II (TaKaRa Bio, USA) made up to a final reaction volume of 10 µL with MilliQ water (Millipore, USA). qPCR was performed under the following conditions: initial denaturation at 94°C for 1 min, 40 cycles of amplification comprising (i) denaturation at 94°C for 10 s, (ii) annealing of primers to template DNA at 60°C for 20 s, and (iii) extension of primers at 72°C for 25 s, followed by a final extension at 72°C for 25 s. Gene expression analysis of the samples in triplicate was performed in real-time.

Histochemical analysis of beta-glucuronidase (GUS) expression was performed as per the protocol (Jefferson et al., 1987; Mahto et al., 2018) GUS reporter system has been used as a selection criterion for transformed plant cells. 100 mg transformed callus was treated with 40 µL GUS extraction buffer containing the substrate 5-bromo-4-chloro-3-indolyl glucuronide (X-Gluc), and the reaction mixture was incubated at 37°C overnight to select transformants with GUS expression. The GUS-stained tissues were observed under a microscope (Olympus IX83).

#### 2.2.3 Camptothecin estimation in cell lines of *N. nimmoniana*

Camptothecin extraction from the transformed and untransformed plant cell biomass was performed as reported by Khwajah Mohinudeen et al. (2021) and Venugopalan et al. (2016).

Approximately 0.3 g of transformed and untransformed dried biomass dissolved in 20 mL of distilled water was homogenized with liquid nitrogen in a pestle-mortar followed by liquid–liquid extraction thrice using 50 ml of chloroform:methanol solvent mixture (4:1). The CPT organic layer was collected and evaporated using a rotary vacuum evaporator (Superfit, India). The dried camptothecin extract was resuspended in 1 mL of DMSO:methanol (1:50) and filtered through a 0.2 µm filter for further analysis. Approximately 20 µL of CPT extract was injected into RP-HPLC (LC-20AD, Shimadzu, Japan) using 25% acetonitrile as the mobile phase at a flow rate of 0.8 ml/min. An ODS Hypersil gold column (Thermo Fisher, USA) with a particle size of 5 µm was used as the stationary phase, with a column temperature of 30 °C. CPT absorbance was measured using a photodiode array detector at 254 nm. The concentration of CPT in the sample was estimated using a standard calibration curve of peak area vs. known concentration of CPT (**Dataset S1**) generated using authentic CPT samples (>90% purity, Sigma Aldrich, USA). The retention time of CPT from the standard was 17.3 min, and the peak area obtained from the plant cell extracts at the same retention time was compared to the standard plot to estimate the corresponding concentration and yield from transformed and untransformed cell lines.

## 3 Results

### 3.1 Reconstruction of the *N. nimmoniana* model NothaGEM

A genome-scale metabolic model for camptothecin production was constructed by incorporating the available genome data into ModelSEED. The draft model comprised 1,162 metabolites and 1,104 reactions, including the central carbon metabolism and tryptophan biosynthesis. The reaction directionality was modified based on information available in the Plant Metabolic Network database (PMN) (https://plantcyc.org/organism-summary?object=CACUMINATA). The nomenclature of the metabolites/identifiers was maintained uniformly from Kyoto Encyclopedia of Genes & Genomes (KEGG).

Twenty camptothecin biosynthesis reactions were added to the model, of which 18 reactions were directly involved in the CPT pathway, one transport reaction of tryptamine from the stroma to the cytosol and one exchange reaction to account for the secretion of camptothecin. Dead-end metabolites and blocked reactions were removed, specifically from the stroma and cytosolic compartments, since biomass production and camptothecin biosynthesis were focused in these two compartments. Some important metabolites, such as beta-carotene, ascorbate, and vitamin E, were detected as dead-ends. As a gap-filling step, the corresponding biosynthetic and metabolic reactions were added to the model. The steps involved in gap filling during manual curation are discussed in **Dataset S1**. Overall, the total blocked reactions were reduced to 299 from 385, and the dead-end metabolites were reduced to 413 from 507. GC–MS analysis of *N. nimmoniana* cells and the suspension culture filtrate revealed the presence of mannitol, malate, and oxo-proline. Hence, the reactions associated with them were incorporated into the model to improve its predictive capability. Stoichiometrically inconsistent reactions were identified and replaced with balanced, consistent reactions to make the model entirely stoichiometrically consistent with mass and charge balance with no duplicate reactions. The final model, NothaGEM *i*SM1809, comprised 1228 metabolites, 1,233 reactions, and 1,809 genes (**Figure 2**).

The model was compartmentalized into 11 compartments, namely, cytosol, stroma, Golgi apparatus, vacuole, cell wall, peroxisome, and mitochondria. There were 23 exchange reactions and 296 transport reactions, indicating high metabolite interconnectivity among intracellular compartments and 914 metabolic reactions. The model properties and list of reactions added to the model are described in **Dataset S2**. Before curation, our model showed a MEMOTE score of 14%. After extensive curation followed by annotation of metabolites and reactions, the current MEMOTE score of our model, NothaGEM is 67%.

### 3.2 Biomass analysis and model validation

The biomass reaction generated by the model was experimentally validated by analyzing the biomass of *N. nimmoniana* cells and the culture filtrate. From the growth kinetics (**Dataset S1**), the maximum biomass growth was observed on the 15th day; hence, samples were collected from the 15th day for further analysis. The total lipid content was estimated to be 3.43 ± 0.19% DW (**Dataset S1**), and the total protein content was 23.15 ± 0.55% DW (**Dataset S1**). To calculate the coefficients of each amino acid incorporated into the biomass equation, the proteomic sequence was used and provided as input to the Protein Information Resource (PIR) database (https://proteininformationresource.org/), which provides the codon usage frequency of each residue. Subsequently, the coefficients were predicted using the individual molecular weight of each amino acid. **Dataset S1** shows how the amino acid coefficients were estimated and how they were similar to the model-predicted coefficients. The experimental coefficients of amino acids were applied to the biomass reaction in the model (Thiele and Palsson, 2010).

The experimental media components included as uptake constraints are mentioned in **Dataset S1**. The metabolites taken up by the plant cells were estimated using the difference between the metabolite concentration on the 15th day and day 0 (as mentioned in **Dataset S1**). The metabolites for which the values could be calculated experimentally were incorporated as the lower bounds in the model. Other metabolite fluxes that could not be calculated were left unconstrained at their default values. We performed FBA with the objective function of maximizing biomass and estimating growth rates. While the experimental specific growth rate was estimated to be 0.0019 h^−1^ (**Dataset S1**), the model predicted a specific growth rate of 0.0014 h^−1^, highlighting the accuracy of the model with respect to the experimental data (**Figure 2C**). A flux map (created with BioRender.com) from sucrose uptake to central carbon metabolism for biomass formation in NothaGEM *i*SM1809 is depicted in **Dataset S1** **Figure 3**, where the thickness of the arrows is proportional to the flux values obtained in the FBA simulation.

### 3.3 *In silico* prediction of metabolic targets for enhanced camptothecin production using constraint-based modeling methods

We used FSEOF to screen and identify overexpression and knockout targets for camptothecin production. Fifty-five reactions were identified and ranked among the overexpression targets using the ‘*f*’ score mentioned in *Section 2.1*. In the case of multiple reactions with the same *f* values, ranking was based on the strategic location of the CPT biosynthetic pathway.

**Table 1A** lists the top 10 enzymatic reaction targets predicted by the model. Based on the *f* score, STR was one of the top-ranked enzymes. STR catalyzes the conversion of tryptamine and secologanin to strictosidine, an important monoterpene indole alkaloid intermediate in camptothecin biosynthesis (**Figure 1**). The main criterion for selection of STR for over-expression was its strategic location, and therefore, it would have a more direct effect on camptothecin biosynthesis. This model predicts STR synthase overexpression was experimentally implemented using *Agrobacterium*-mediated transformation. Among the other top-ranked targets predicted, CPT biosynthesis pathway reactions, including the mevalonic acid and shikimate pathways, amino acid metabolism, methionine, and nucleotide biosynthesis, were also predicted but not experimentally validated. The model predicted some new leads for overexpression targets such as succinate and citrulline (**Table 1A**). This is concurrent with the literature, where succinic acid has been used to enhance terpene alkaloid production three-fold in *Catharanthus roseus* (Changxing et al., 2020).

**Table 1:** (A) Top-ranked overexpression targets predicted for enhancing CPT production, (B) Top-ranked knockout targets predicted for enhancing CPT production.

Rank	Enzymes catalyzing the target reactions	Metabolic pathway- Key product – Reaction identifier*	*f* Score
A			
1	Strictosidine synthase	CPT biosynthesis – Strictosidine – rxn02655_c0	1
2	Tryptophan decarboxylase	Shikimate pathway – Tryptamine – rxn00484_d0	1
3	Geraniol 10 hydroxylase	MVA pathway – Geraniol – rxn16769_c0	1
4	Tryptophan synthase	Shikimate pathway – Indole – R_rxn28615_d0	1
5	Mevalonate oxidoreductase	MVA pathway – Mevalonic acid – R_rxn01501_c0	0.53
6	Adenosine kinase	Nucleotide biosynthesis – Adenosine diphosphate - R_rxn00134_c0	0.53
7	Homocysteine hydrolase	Amino acid biosynthesis – Citrulline – R_rxn01019_d0	0.53
8	Citrulline Aspartate Ligase	Amino acid biosynthesis – Argininosuccinate – R_rxn01434_d0	0.22
9	Succinate CoA ligase	TCA cycle – Succinate – R_rxn10952_m0	0.15
10	5-Oxoprolinase	Vitamin biosynthesis - 5-Oxoproline – rxn00186_c0	0.13
Rank	Enzymes catalyzing target reactions	Metabolic pathway- Key product – Reaction identifier	*f* Score
B			
1	Phosphoserine phosphohydrolase	Amino acid biosynthesis - Phosphoserine - R_rxn00420_d0	9.8
2	3-Phosphoglycerate oxidoreductase	CPT biosynthesis - 3-Phosphonooxypyruvate - R_rxn01101_d0	9.8
3	Methenyl tetrahydrofolate hydrolase	Co-factor biosynthesis - Methenyltetrahydrofolate - R_rxn01211_c0	8.6
4	2-Phosphoglycerate phosphomutase	CPT biosynthesis - 2-Phospho-D-glycerate - R_rxn01106_c0	8.1
5	ATP Citrate lyase	TCA cycle - Citrate - ‘R_rxn00257_c0’	6.5
6	Methylenetetrahydrofolate oxidoreductase	Co-factor biosynthesis - 5-10-Methylenetetrahydrofolate - R_rxn00907_d0	5.6
7	Glyceraldehyde 3-phosphate ketose isomerase	CPT biosynthesis - Glycerone-phosphate - R_rxn00747_d0	4.9
8	Malate NAD+ oxidoreductase	TCA cycle - Malate - R_rxn00248_c0	2.8
9	Glutamate oxidoreductase	Amino acid biosynthesis - Glutamate – R_rxn00184_c0	2.6
10	Phosphoglycerate hydrolase	CPT biosynthesis – Phosphoenolpyruvate - R_rxn00459_d0	2.0

*Reaction identifier c0 represents the reaction target predicted belongs to the cytoplasm, d0 represents the reaction target in the stroma.

Among the knock-out targets predicted, 24 reactions were identified and ranked using the score ‘*f*’ mentioned earlier. **Table 1B** lists the top 10 enzymatic reaction targets predicted by the model. Among the top-ranked targets for knock-outs, reactions involving the formation of folates, serine, and conversion of acetyl CoA and oxaloacetate to malate and citrate have been predicted. Isomerization reactions involving the conversion of 3-phosphorglycerate to phosphoenolpyruvate and glyceraldehyde-3-phosphate to glycerophosphate have also been predicted.

### 3.4 Development of metabolically engineering cell lines of *N. nimmoniana*

**Figure 4:**
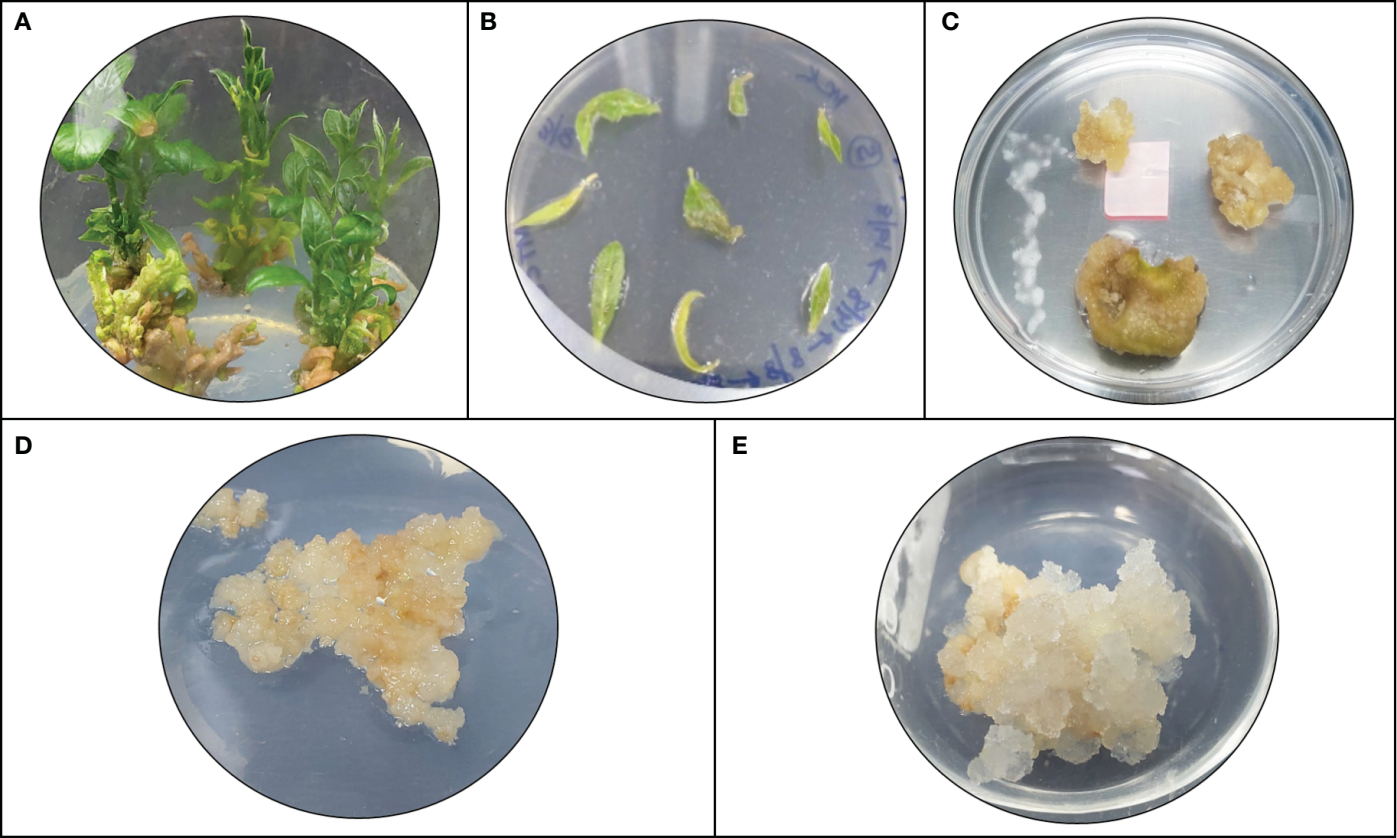
Plant cell/tissue cultures of *N.* nimmoniana; **(A)** *In vitro* shoots of *N. nimmoniana*; **(B)** *in vitro* leaf explants after *Agrobacterium* mediated transformation; **(C)** initiation of callus from leaf explants; **(D)** wildtype callus; **(E)** transgenic callus harboring overexpressed At-STR gene.

**Figure 5:**
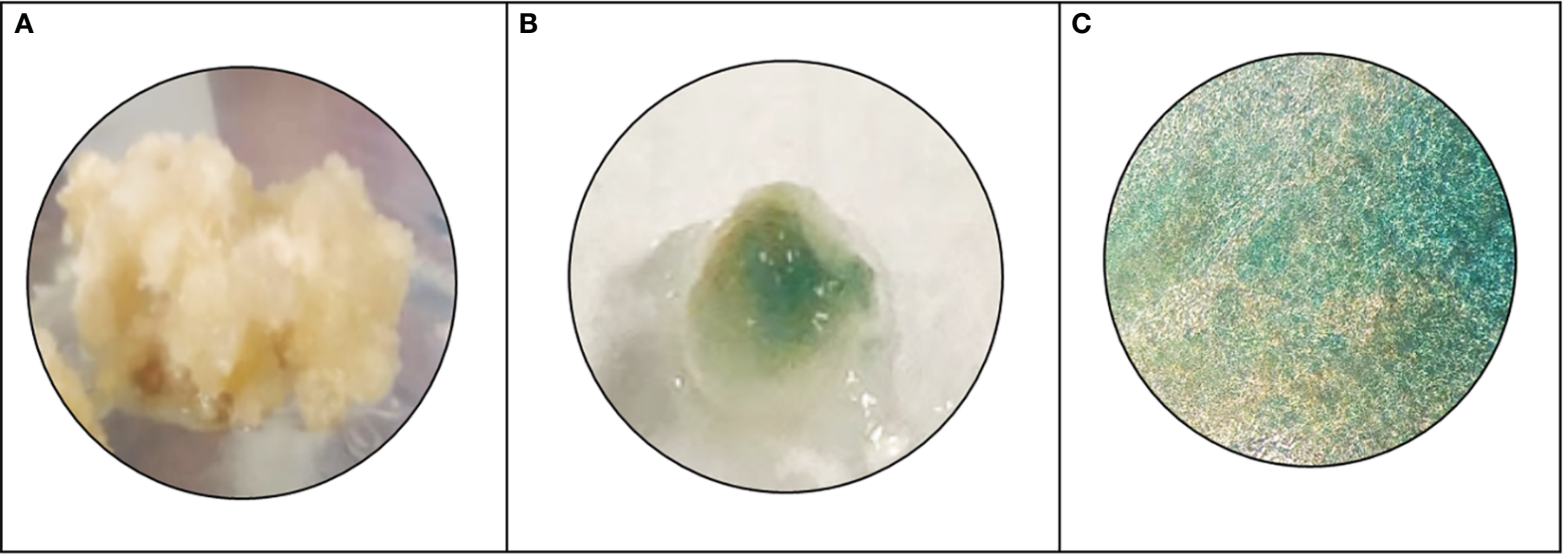
Histochemical GUS analysis, **(A)** wild type *N. nimmoniana* callus, **(B)** verification of GUS expression as indicated by blue coloration of the transformed callus of *N. nimmoniana*, **(C)** GUS expression observed under ×5 optical microscope.

**Figure 6:**
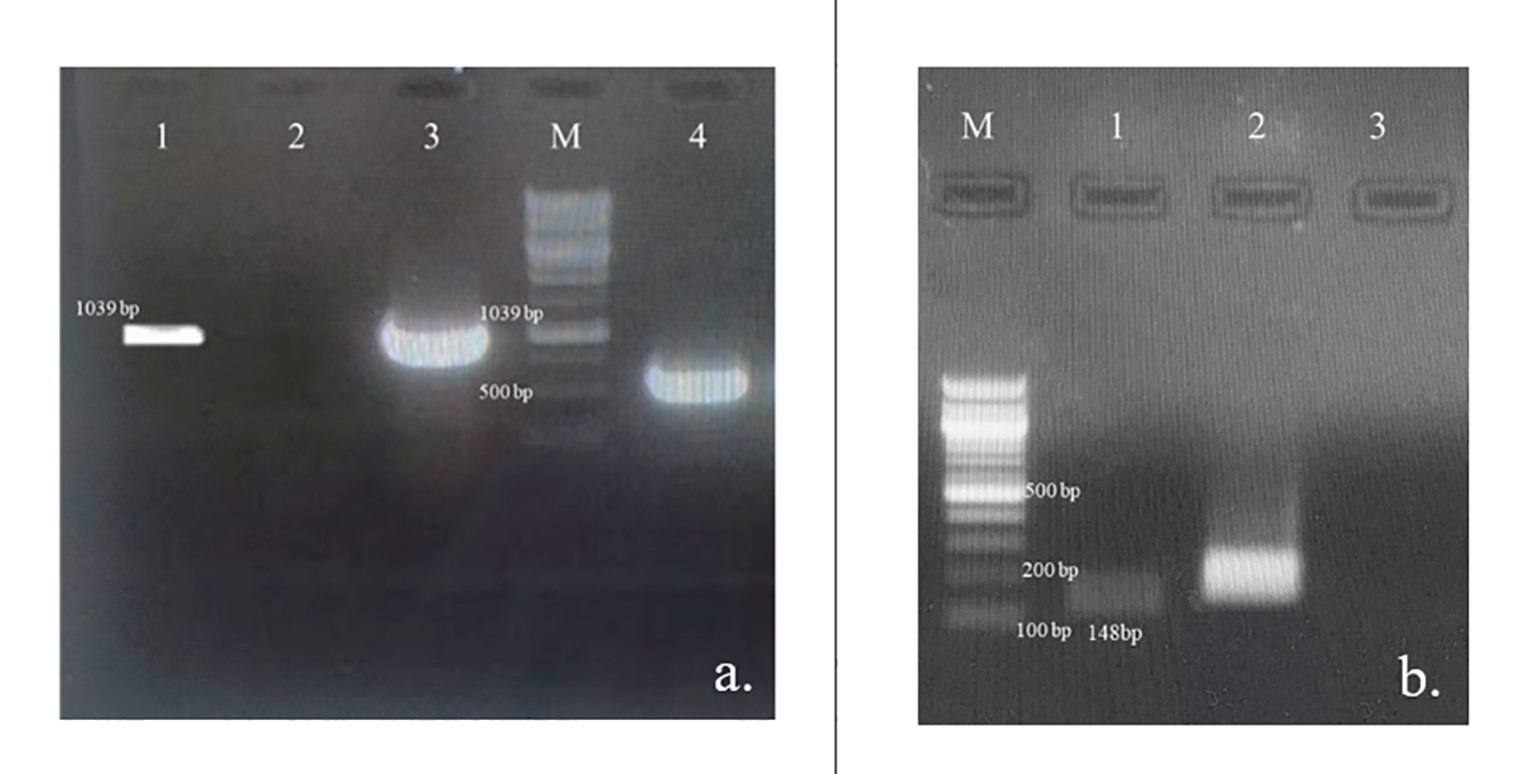
Verification of the At-STR gene integration and expression; **(A)** PCR amplification of At-STR gene (1,039 bp) from the transformed cell lines of *N. nimmoniana* visualized on 0.8% agarose gel. Lane 1: plasmid DNA of A. tumefaciens LBA4404 harboring At-STR construct of 1,039 bp (positiv control), Lane 2: Untransformed cell line, WN1 (negative control), Lane 3: PCR product of transformed cell line (STR2) showing amplification corresponding to At-STR (1,039 bp), Lane M: 1 Kb ladder, Lane 4: PCR product (STR2) showing GUS expression (500 bp); **(B)** Verification of At-STR transgenic expression in untransformed and transformed cell line STR2 using semi-quantitative RT-PCR followed by visualization of the amplified products on a 1% agarose gel Lane M: 100 bp ladder, Lane 1: At-STR expression in *N. nimmoniana* (148 bp), Lane 2: Nn-Actin (Positive internal control), Lane 3: Water (negative control).

The model predicted that the top-ranked target STR could be used to generate engineered cell lines of *N. nimmoniana. In vitro-*grown shoots, leaves, and petiole regions of *N. nimmoniana* were transformed using *A. tumefaciens* LBA4404 with pCAMBIA 1301 containing the At-STR cDNA. Fifteen transformed cell lines were induced after four months of transformation, of which four cell lines (**Figure 4**) grew sustainably after 10 subcultures. Transgenic expression was confirmed using GUS analysis (**Figure 5**), PCR, and qPCR (**Figure 6**).

### 3.5 Camptothecin estimation from over-expressed cell lines of *N. nimmoniana*

**Figure 7:**
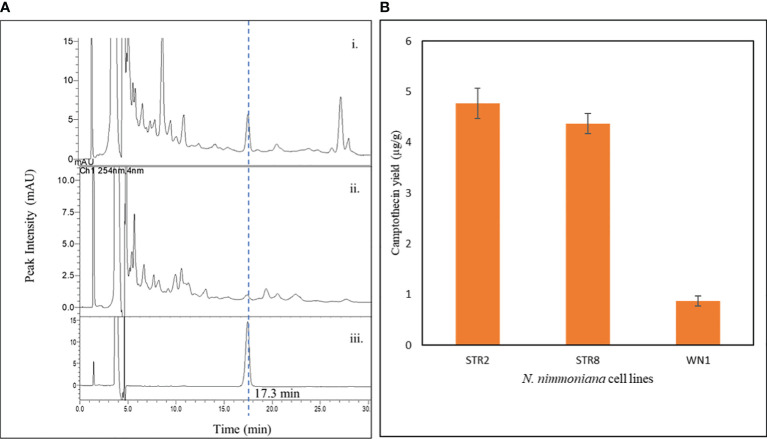
**(A)** HPLC chromatogram of CPT detected in various samples—i. Metabolically engineered *N. nimmoniana* callus harboring At-STR; ii. Wild-type *N. nimmoniana* callus; iii. Standard Camptothecin; **(B)** Camptothecin yield from callus cultures of *N. nimmoniana.* STR, strictosidine synthase callus, WN, Wildtype *N. nimmoniana* callus.

CPT was extracted from the transformed and untransformed cell biomass and estimated using RP-HPLC. **Figure 7** shows the CPT chromatogram and yield obtained from the cell lines of *N. nimmoniana* from HPLC analysis. CPT yield of 4.77 µg g^−1^, 4.37 µg g^−1^, and 0.87 µg g^−1^ were obtained from *N. nimmoniana* over-expressed cell lines STR2, STR8, and the wild-type cell line (WN1), respectively.

## 4 Discussion

Increasing market demand for camptothecin production has led to excessive deforestation, endangering *N. nimmoniana* in India. Additionally, as a tree species, it has a slow growth rate and typically requires 7–8 years before it can be harvested for the first time. Alternative methods for the *in vitro* production of camptothecin in bioreactors using plant cell suspensions and the development of high-yielding plant varieties are needed to prevent the depletion of natural sources that will be efficient, eco-friendly, and cost-effective to sustainably produce CPT on a large scale. In this regard, rationally designed metabolic engineering strategies can be used to generate high-yielding plant cell lines.

Therefore, we reconstructed a genome-scale metabolic model for CPT production. The draft model generated from ModelSEED represents different metabolic pathways in plants, including the central carbon metabolism. Since sucrose was the primary carbon source used to grow *N. nimmoniana*, our initial steps involved analyzing the model to produce biomass, stepwise from sucrose, and other media components. Although we could account for biomass production, the production of CPT was also an essential objective of our work. To achieve this, we added reactions corresponding to CPT biosynthesis involving the shikimic acid pathway, mevalonic acid pathway, and the methylerythritol pyrophosphate pathway systematically in their respective compartments, as reported by Rather et al. (2018) and Murali et al. (2021). While performing FBA, we observed that an increase in biomass flux led to a decrease in camptothecin flux, and vice-versa. This demonstrated that camptothecin is a secondary metabolite, increasing the confidence in the predictive power of the drafted metabolic model.

To our knowledge, this is the first report of a metabolic model developed to produce camptothecin. CPT is produced from strictosidine, which is a crucial intermediate in plant indole alkaloid biosynthesis. This model can be extended to produce other commercially important and pharmaceutically valuable phytochemicals that can be synthesized from strictosidine. Some well-known secondary metabolites produced from strictosidine include quinine, which is commonly used to treat malaria and babesiosis, serpentine and ajmalicine, which are used to treat hypertension, and vinca alkaloids such as vincristine and vinblastine used in anti-cancer therapeutics. Using model-predicted strategies, high-yielding plants and cell culture systems can be generated for the *in vitro* production of metabolites in a large scale in bioreactors.

Among the top-ranked predicted overexpression targets, the CPT biosynthesis pathway genes included STR, tryptophan decarboxylase (TDC), geraniol 10 hydroxylase (G10H), tryptophan synthase, and mevalonate oxidoreductase. Overexpression of TDC and STR individually and synergistically has been reported in *C. roseus* by Canel et al. (1998), where STR overexpression showed 10-fold higher STR activity than wild-type cultures. Similarly, the overexpression of G10H in *C. roseus* hairy roots has been reported to enhance catharanthine production (Wang et al., 2010). The co-overexpression of G10H and STR reportedly improves CPT content in the hairy roots of *Ophiorrhiza pumila* (Cui et al., 2015). STR and G10H from *C. roseus* were individually and simultaneously introduced into *O. pumila*, respectively. Overexpression of G10H significantly improved CPT production compared to non-transgenic hairy root culture, implying that G10H plays a vital role in CPT accumulation in *O. pumila.* These studies have also shown a significant increase in CPT production where co-overexpression of G10H and STR demonstrated an increase in the yield of CPT by 56% as compared to single overexpression lines and non-transgenic lines (Cui et al., 2015).

Some novel reactions that are not involved in upstream CPT biosynthesis were also predicted by our approach as targets for overexpression. These targets include reactions involving production of homocysteine, succinate, citrulline, and 5-oxoproline. Homocysteine is known to produce methionine, a vital amino acid that initiates protein synthesis. Homocysteine metabolism, predicted as one of the overexpression targets, has also been reported to be influenced by environmental stress, which can presumably enhance secondary metabolism (Watanabe et al., 2021). Succinic acid, another predicted target, has been used to enhance terpene alkaloid production in *C. roseus*. In a study by Changxing et al., the addition of 10 mM succinic acid as a precursor to *C. roseus* callus cultures enhanced serpentine and ajmalicine production by four and five fold, respectively (Zhao et al., 2001; Changxing et al., 2020). Citrulline, a critical intermediate in the arginine pathway, and 5-Oxoproline producing glutamate were also predicted as CPT overexpression targets. Under stress conditions, the synthesis of abundant amino acids such as proline, glutamate, arginine, and asparagine is upregulated. These amino acids act as compatible osmolytes and precursors for secondary metabolite production (Song et al., 2020), and hence can be used to increase CPT production.

Among the predicted knockout targets, 24 reactions were identified, as shown in **Table 1B**. Two reactions involving the formation of folates, methyl tetrahydrofolate hydrolase and methylenetetrahydrofolate oxidoreductase, were predicted to be knockout targets. While it is well known that folate plays a vital role as a cofactor, it is intriguing to note that it is produced from the shikimate pathway, where chorismate is converted to p-aminobenzoate, which proceeds to synthesize folate (Kołton et al., 2022). When this branched pathway is blocked, it can lead to increased amounts of chorismate, resulting in an increase in CPT production. Similarly, the conversion of serine to phosphoserine was identified as a knockout target. Serine plays an important role in the conversion of indole to tryptophan in CPT biosynthesis (**Figure 1**). Downregulating the conversion of serine to phosphoserine can lead to the enhanced production of tryptophan and, thereby, CPT.

Reactions involving the conversion of oxaloacetate and acetyl-CoA to citrate and malate, respectively, have also been predicted as knockout targets. Acetyl-CoA is a crucial metabolite in the MVA pathway (**Figure 1**). Malate and citrate can be produced *via* other pathways such as the TCA cycle. Downregulation of these reactions can also augment CPT production. Isomerization reactions involving the conversion of 3-phosphoglycerate to phosphoenolpyruvate and glyceraldehyde-3-phosphate to glycerophosphate have also been predicted. 3-phosphoglycerate and G3P are directly involved in the upstream biosynthesis of CPT. Hence, if the steps involving their isomerization can be downregulated, they can be used as rationally predicted metabolic engineering strategies for multi-fold CPT enhancement.

To our knowledge, this is the first report of enhanced camptothecin production from a metabolic engineering strategy in *N. nimmoniana* callus cultures, where a five-fold increase in CPT production was obtained. CPT production was confirmed by HPLC, and its overexpression was confirmed by PCR and qRT-PCR.

## 5 Conclusion

In this study, we successfully enhanced camptothecin yield in *N. nimmoniana* plant cells using a model-driven metabolic engineering strategy. A genome-scale metabolic network, NothaGEM *i*SM1809, was reconstructed for camptothecin production in *N. nimmoniana* cells and curated by integrating the experimental data. *In silico* constraint-based modeling approaches were used to predict strategies for the further enhancement of CPT. Similar to the STR-mediated overexpression strategy, other predicted enzyme targets can be experimentally implemented to enhance CPT production. The prediction of non-intuitive targets, such as citrulline and succinic acid, indicated the predictive power of the model. These targets can also be experimentally implemented in combination or alone to further enhance in CPT yield. To develop a sustainable plant cell-based bioprocess for large-scale *in vitro* production of CPT, additional experiments, such as random amplified polymorphic DNA(RAPD) PCR and restriction fragment length polymorphism (RFLP), can be applied to select the most stable callus lines for process optimization and scale-up in bioreactors.

## Data availability statement

The datasets presented in this study can be found in online repositories. The names of the repository/repositories and accession number(s) can be found in the article/**Supplementary Material**.

## Author contributions

SaM designed and implemented the experiments, analyzed and compiled the results and formulated the manuscript. SmS, being the principal investigator, conceptualized and designed the entire study and contributed to data analysis and manuscript preparation. MI and KR designed the computational methodology and helped with the data analysis. HR performed the growth kinetics and residual sucrose estimation experiments. ShS and ShM assisted with biomass analysis of the samples and provided vital inputs for the study. All authors contributed to the article and approved the submitted version.

## Supplementary material

Supplementary Data Sheet 1 BLASTP analysis of enzymes between *C. acuminata* and *N. nimmoniana.* Collinearity existing between *N. nimmoniana* and *C. acuminata* CPT producing genes; – Steps involved in identifying over-expression and knock-out targets for enhanced CPT production; formulation of the *Nothapodytes nimmoniana* specific biomass reaction comprising the biomass precursors and coefficients, experimental growth kinetics, total protein and lipid estimation, estimation of media components and determination of uptake constraints, standard curve of camptothecin obtained by plotting various concentrations of camptothecin against their respective peak area from HPLC, list of oligonucleotide primers used for amplification.

Supplementary Data Sheet 2 NothaGEM model parameters, comprising - S2.1 – Metabolites, S2.2 – Reactions, S2.3 –Memote report, S2.4 – Model (SBML file).
