# Supplementary material for: Genome-scale metabolic model led engineering of Nothapodytes nimmoniana plant cells for high camptothecin production
Source: Front Plant Sci. 2023 Aug 2;14:1207218. doi: 10.3389/fpls.2023.1207218 (PMC10433906; doi:10.3389/fpls.2023.1207218)
Supplement: Supplementary Data Sheet 1 — BLASTP analysis of enzymes between C. acuminata and N. nimmoniana. Collinearity existing between N. nimmoniana and C. acuminata CPT producing genes; – Steps involved in identifying over-expression and knock-out targets for enhanced CPT production; formulation of the Nothapodytes nimmoniana specific biomass reaction comprising the biomass precursors and coefficients, experimental growth kinetics, total protein and lipid estimation, estimation of media components and determination of uptake constraints, standard curve of camptothecin obtained by plotting various concentrations of camptothecin against their respective peak area from HPLC, list of oligonucleotide primers used for amplification. [file DataSheet_1.docx]

Supplementary Material

**Genome-scale metabolic model led engineering of *Nothapodytes nimmoniana* plant cells for high camptothecin production**

Sarayu Murali ^1^, Maziya Ibrahim ^1,2,3^, Hemalatha Rajendran ^1^, Shagun Shagun ^4^,

Shyam Kumar Masakapalli ^4^, Karthik Raman ^1,2,3^, Smita Srivastava ^1*^

^1^Department of Biotechnology, Bhupat & Jyoti Mehta School of Biosciences, Indian Institute of Technology Madras, Chennai, 600 036, India

^2^Initiative for Biological Systems Engineering (IBSE), Indian Institute of Technology Madras, Chennai, 600 036, India

^3^Robert Bosch Centre for Data Science and Artificial Intelligence (RBC-DSAI), Indian Institute of Technology Madras, Chennai, 600 036, India

^4^School of Biosciences and Bioengineering, Indian Institute of Technology Mandi – 175 005, Himachal Pradesh, India

***Correspondence:**

Smita Srivastava

smita@iitm.ac.in

# Supplementary Data


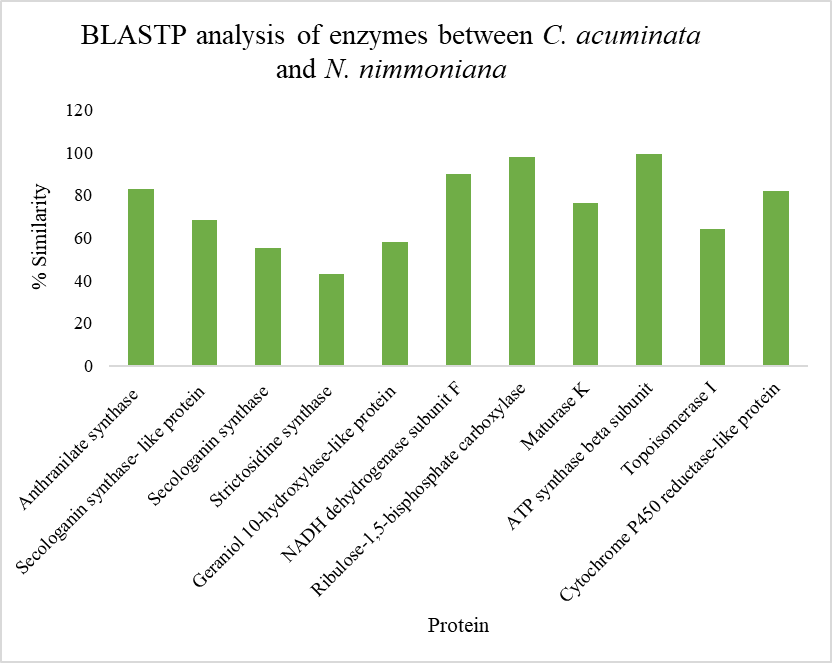


Fig. 1 – BLASTP analysis of enzymes between *C. acuminata* and *N. nimmoniana.* Collinearity existing between *N. nimmoniana* and *C. acuminata* CPT producing genes.


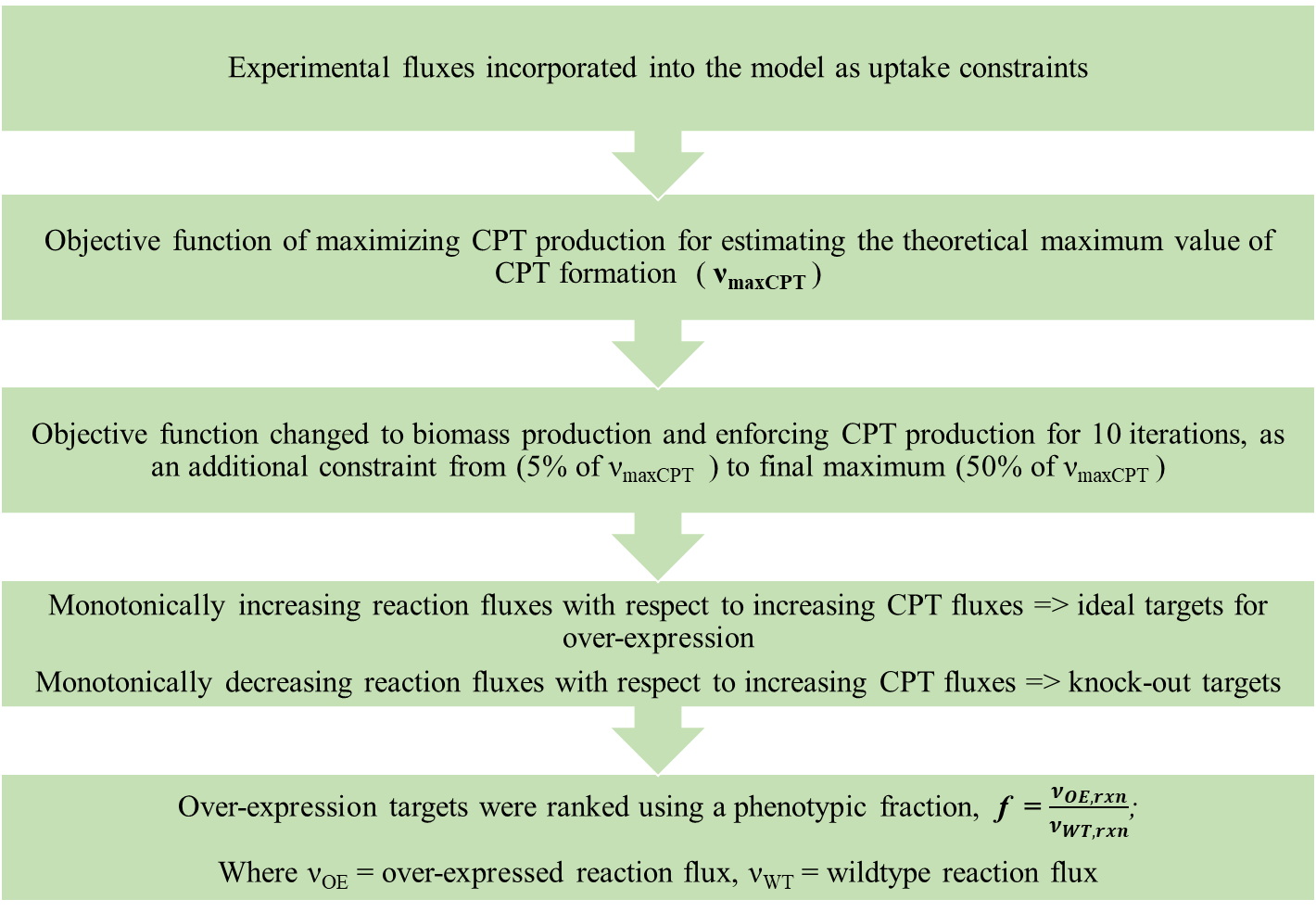


Fig. 2 – Steps involved in identifying over-expression and knock-out targets for enhanced CPT production


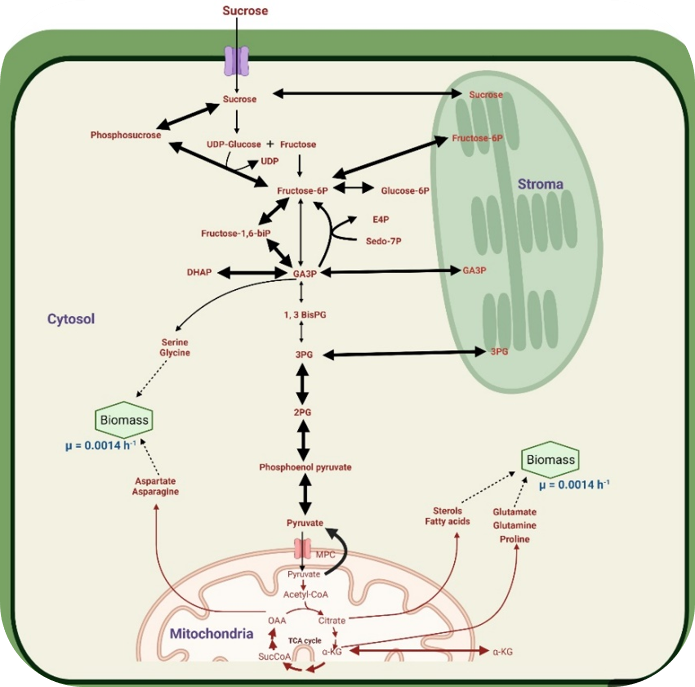


Fig. 3 – The central carbon metabolism of NothaGEM *i*SM1809 leading to biomass formation depicted through a metabolic flux-map. Abbreviations – Fructose-6P – fructose 6-phosphate, Glucose-6P – glucose 6-phosphate, E4P – erythrose 4-phosphate, Sedo-7P – sedoheptulose 7-phosphate, DHAP – dihydroxyacetone phosphate, GA3P – glyceraldehyde 3-phosphate, 1,3 BisPG – 1,3 bisphosphoglycerate, 3-PG – 3-phosphoglycerate, 2PG – 2-phosphoglycerate, MPC – mitochondrial pyruvate carrier, OAA – oxaloacetate, SucCoA- succinyl CoA, αKG – alpha-ketoglutarate

**Formulation of the *Nothapodytes nimmoniana* specific biomass reaction**

**Abstract**

The current report describes the methods used to generate a biochemical reaction equation for the biosynthesis of *N. nimmoniana* specific biomass. The equation gave an idea about the biochemical composition of *N. nimmoniana* cells and was used to generate the biomass objective function (BOF) for the genome-scale metabolic model NothaGEM using constraint-based methods (Feist and Palsson 2010).

**Culture conditions**

*N. nimmoniana* cell suspensions were initiated from *N. nimmoniana* callus cultures grown in MS medium with an initial pH of 5.8 and incubated at 23 ℃ with a 16/8 h photoperiod. The cell suspension was subcultured every month. The cell biomass and the culture filtrate were lyophilized and subsequently used for analysis.

**Growth Kinetics**

A growth kinetics (Fig. 2) was performed to determine the growth of *N. nimmoniana* cells. From the experiment, 15^th^ day cultures were taken for biomass analysis. The experimental specific growth rate was obtained from the slope of the graph plotted between biomass(logarithmic) vs time and estimated to be 0.0019 h^-1^. Residual sucrose estimation was performed in a High-performance liquid chromatography (Agilent Technologies, USA) using a refractive index detector (RID) to determine the amount of substrate utilized.


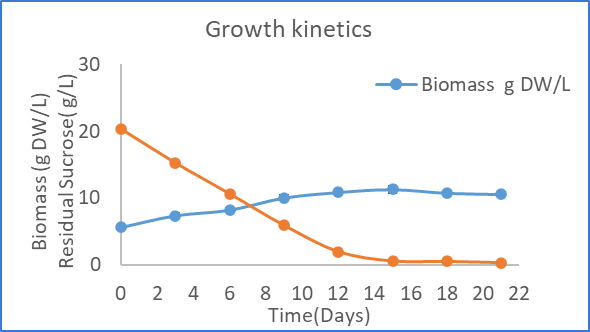


Fig. 4 Growth kinetics of *N. nimmoniana* plant cells

**Biomass composition**

The biomass precursors were divided into major categories – protein, nucleic acid carbohydrates, lipids, cell wall components, inorganic ions, and metabolites.

**Proteins**

Extraction of the total proteins present in the *N. nimmoniana* lyophilized plant material was performed using an extraction buffer. Lyophilized plant cells (5 mg) were extracted in 500 µl of the extraction buffer composed of 125 mM Tris-HCl (pH-8.8), 1% Sodium dodecyl sulfate (SDS; w/v), 10% glycerol (v/v) and 50 mM Sodium metabisulfite (Na_2_S_2_O_5_) and immediately transferred to ice for ~30 min. After preparing the extracts, eppendorf tubes were removed from ice and kept at room temperature for the re-solubilization of SDS. The extracts were centrifuged at 13,000g for 10 min and the supernatant was collected to fresh eppendorf tubes for further proteins estimation using Bio-Rad DC (detergent compatible) protein assay. Different concentrations (0, 0.3, 0.6, 0.9, 1.2 and 1.5 mg/mL) of the protein standard BSA (bovine serum albumin) was prepared by using the same extraction buffer for the quantification of proteins in the sample. 0.005 ml of the different concentrations of the standard proteins and plant extracts (4 replicates) were added into microplate wells (Fig. 3) 0.025 ml of working reagent A [20 µl of reagent S mixed in 1 ml of reagent A (alkaline copper tartrate solution)] was added to wells followed by 0.2 ml of reagent B (dilute folin reagent). After 15 minutes incubation, absorbance was measured at 750 nm in the microplate reader (Martínez‐García et al., 1999). The total protein was 23.15 ± 0.55 %DW.

To calculate the coefficients of each amino acid to be incorporated into the biomass equation, the proteomic sequence was input to the PIR database and the frequency of each residue was calculated. Subsequently, using the individual molecular weights of each amino acid, the coefficient was predicted as mentioned in (Thiele and Palsson 2010). Table 1a and 1b ~~2a and 2b~~ show how the coefficients of amino acids were estimated. The model predicted coefficients and the experimentally predicted coefficients were observed to be similar and have been highlighted. The experimental coefficients of the amino acids were applied to the biomass reaction in the model.

| Triple letter code | Single letter code | Amino acid | Mol weight (g/mol) | Residues in proteome | %age of monomer per genome (mol/mol) | g monomer/ mol macromolecule (g/mol) | g monomer/ g macromolecule (g/g) | mmol monomer/ g DW (mmol/g DW) |
| --- | --- | --- | --- | --- | --- | --- | --- | --- |
| Ala | A | Alanine | 89.09 | 1110931 | 0.066 | 5.875 | 0.045 | 0.117 |
| Cys | C | Cysteine | 121.15 | 322220 | 0.019 | 2.317 | 0.018 | 0.034 |
| Asp | D | Aspartic acid | 133.1 | 877151 | 0.052 | 6.930 | 0.054 | 0.092 |
| Glu | E | Glutamic acid | 147.13 | 1079182 | 0.064 | 9.425 | 0.073 | 0.114 |
| Phe | F | Phenylalanine | 165.19 | 716386 | 0.043 | 7.024 | 0.054 | 0.076 |
| Gly | G | Glycine | 75.07 | 1119222 | 0.066 | 4.987 | 0.039 | 0.118 |
| His | H | Histidine | 155.16 | 409376 | 0.024 | 3.770 | 0.029 | 0.043 |
| Ile | I | Isoleucine | 131.17 | 920900 | 0.055 | 7.170 | 0.055 | 0.097 |
| Lys | K | Lysine | 146.19 | 998379 | 0.059 | 8.663 | 0.067 | 0.105 |
| Leu | L | Leucine | 131.17 | 1637850 | 0.097 | 12.752 | 0.098 | 0.173 |
| Met | M | Methionine | 149.21 | 411871 | 0.024 | 3.648 | 0.028 | 0.043 |
| Asn | N | Asparagine | 132.12 | 759564 | 0.045 | 5.957 | 0.046 | 0.080 |
| Pro | P | Proline | 115.13 | 833546 | 0.049 | 5.696 | 0.044 | 0.088 |
| Gln | Q | Glutamine | 146.15 | 632095 | 0.038 | 5.483 | 0.042 | 0.067 |
| Arg | R | Arginine | 174.2 | 891960 | 0.053 | 9.223 | 0.071 | 0.094 |
| Ser | S | Serine | 105.09 | 1541708 | 0.092 | 9.617 | 0.074 | 0.163 |
| Thr | T | Threonine | 119.12 | 821016 | 0.049 | 5.805 | 0.045 | 0.087 |
| Val | V | Valine | 117.15 | 1082923 | 0.064 | 7.530 | 0.058 | 0.114 |
| Trp | W | Tryptophan | 204.23 | 217545 | 0.013 | 2.637 | 0.020 | 0.023 |
| Tyr | Y | Tyrosine | 181.19 | 463564 | 0.028 | 4.986 | 0.039 | 0.049 |
|  |  |  |  |  |  |  |  |  |
|  |  | **Total residues (PIR) = 16847389** | | | **Sum of all AA weights (g/mol) =** | **129.494** |  |  |

Table 1a. Estimation of Amino acid coefficients

| **Triple letter code** | **Single letter code** | **Amino acid** | **Model predicted flux**  **(mmol gDW^-1^h^-1^)** | **Experimental flux**  **(mmol gDW^-1^h^-1^)** |
| --- | --- | --- | --- | --- |
| Ala | A | Alanine | 0.161 | 0.117 |
| Cys | C | Cysteine | 0.029 | 0.034 |
| Asp | D | **Aspartic acid** | **0.098** | **0.092** |
| Glu | E | Glutamic acid | 0.130 | 0.114 |
| Phe | F | **Phenylalanine** | **0.074** | **0.076** |
| Gly | G | Glycine | 0.150 | 0.118 |
| His | H | Histidine | 0.038 | 0.043 |
| Ile | I | Isoleucine | 0.083 | 0.097 |
| Lys | K | Lysine | 0.096 | 0.105 |
| Leu | L | **Leucine** | **0.172** | **0.173** |
| Met | M | Methionine | 0.049 | 0.043 |
| Asn | N | Asparagine | 0.056 | 0.080 |
| Pro | P | Proline | 0.096 | 0.088 |
| Gln | Q | Glutamine | 0.071 | 0.067 |
| Arg | R | Arginine | 0.110 | 0.094 |
| Ser | S | Serine | 0.100 | 0.163 |
| Thr | T | Threonine | 0.096 | 0.087 |
| Val | V | Valine | 0.127 | 0.114 |
| Trp | W | **Tryptophan** | **0.024** | **0.023** |
| Tyr | Y | **Tyrosine** | **0.049** | **0.049** |

Table 1b. Estimation of Amino acid coefficients

**Lipids**

Total lipids from the lyophilized plant cells were extracted and quantified by using Bligh and Dyer method with some modifications. Lyophilized plant cells (30 mg) were taken in eppendorf tubes and 3 ml chloroform: methanol (1:2) was added. The extracts were centrifuged at 3,000 rpm for 5 minutes and supernatant was collected to fresh eppendorf tubes. The pellets were re-extracted using 3 ml of chloroform: methanol (1:2) and 0.8 ml of 1% KCl. The extracts were centrifuged again at 3,000 rpm for 5 minutes and supernatant was collected again. To the collected supernatant, 2 ml of chloroform and 1.2 ml of 1% KCl was added and vortexed well. The lipid containing lower layer was then transferred to the fresh previously weighed eppendorf tubes and left for evaporation. After drying, the remaining content was measured represents the total lipids present (Bligh et al., 1959). The total lipid content was to be 3.43 ± 0.19 % DW..

**Extraction of plant metabolites**

For the extraction of metabolites present in *Nothapodytes nimmoniana* plant cells, 20 mg lyophilized sample was extracted in 940 µl extraction solvent composed of 3:1:1 methanol: chloroform: water (v/v/v). 60 µl of Ribitol (0.2 mg/ml) was added as an internal standard followed by heating at 70 °C for 5 minutes at 950 rpm. The extract was centrifuged at 13,000 × g for 10 minutes at room temperature. After centrifugation, the supernatant was transferred to a new fresh centrifuge tube. For the extraction of metabolites from the suspension cells, 50 µl methanol with internal standard (Ribitol; 0.02%) were added to the lyophilized suspension cells. The extract was centrifuged at 13,000 × g for 10 minutes at room temperature and supernatant was transferred to fresh centrifuge tube. Suitable aliquots from each supernatant were taken and dried under-speed vacuum evaporator and derivatized using MeOX-TMS (methoxamine-trimethyl silane) derivatisation. The derivatised samples were centrifuged at 13,000 g for 10 minutes and supernatant was transferred to glass vial for GC-MS analysis (Lisec et al., 2006; Lingwan et al., 2021; Lingwan et al., 2022).

**Gas Chromatography-Mass Spectrometry analysis and metabolites identification**

GC-MS data acquisition was performed on an Agilent instrument with HP-5ms column. Injection volume was 1 µl and helium (carrier gas) flow was 0.6 ml/min. The oven temperature gradient was set to 50 °C and then raised to 70 °C in 5 min hold time ~~for 4 min~~. Further, ramped to a final 200 °C and 300 °C temperature at 10 °C/min and 5 °C/min with 10 min hold time. The raw GC-MS spectra were baseline corrected by using MetAlign software (Lommen et al., 2012). The metabolites identification was done using libraries NIST17 (National Institute of Standards and Technology, Maryland), Fiehn13 (Agilent Technologies, USA) and commercial standards available. For each metabolite, retention times, m/z fragmentation patterns and probability score were extracted (Masakapalli et al., 2014).

**Nucleic acids, Ammonia and Nitrate Estimation**

The total nucleotides (DNA and RNA) were extracted using Genetix’s DNASure Plant Mini kit and Qiagen RNAEasy Plant Mini kit respectively, using the manufacturer’s protocol and were quantified using a Nanodrop (Thermo Fisher Scientific, USA). Ammonia and nitrate content were calculated using spectrophotometric assays – Berthelot’s reaction (Solorzano 1969) and salicylic acid method (Cataldo et al. 2008), where ammonium sulphate and potassium nitrate were used as standards respectively. Elemental ions – Potassium, Calcium, Magnesium, Sodium, and Ferrous ion contents were estimated using the inductively coupled plasma optical emission spectrometry (ICP-OES, Perkin Elmer Optima 5300 DV) facility.

**Uptake constraints**

Experimental media components were included to the model as uptake constraints. Defined media composition of Gamborg’s B5 is mentioned in Table 2a ~~4a~~ and the values of the estimated metabolites taken up by the plant cells (difference between the metabolite concentration on 15^th^ day and 0^th^ day) have been mentioned in Table 2b ~~4b~~.The metabolites for which the values could be calculated experimentally in this section (highlighted) were incorporated as lower bounds into the model and the other metabolites who’s values could not be calculated were left unconstrained at their default values.

| **Media Composition** | | |
| --- | --- | --- |
|  | **Concentration**  **(mg/L)** | **Molecular weight (g/mol)** |
| **Macroelements** | | |
| Sucrose | 30000.00 | 342.30 |
| Ammonium sulphate | 134.00 | 132.14 |
| Calcium chloride | 113.32 | 110.98 |
| Magnesium sulphate | 122.09 | 120.37 |
| Potassium nitrate | 2500.00 | 101.10 |
| Sodium phosphate monobasic | 130.42 | 119.98 |
| **Microelements** | | |
| Boric acid | 3.00 | 61.84 |
| Cobalt chloride hexahydrate | 0.03 | 237.93 |
| Copper sulphate pentahydrate | 0.03 | 249.69 |
| EDTA disodium salt dihydrate | 37.30 | 372.24 |
| Ferrous sulphate heptahydrate | 27.80 | 278.02 |
| Manganese sulphate monohydrate | 10.00 | 169.02 |
| Molybdic acid (sodium salt) | 0.21 | 190.00 |
| Potassium iodide | 0.75 | 166.00 |
| Zinc sulphate heptahydrate | 2.00 | 287.60 |
| **Vitamins** | | |
| myo-Inositol | 100.00 | 180.16 |
| Nicotinic acid | 1.00 | 123.11 |
| Pyridoxine HCl | 1.00 | 205.64 |
| Thiamine hydrochloride | 10.00 | 337.30 |

Table 2a. Defined media composition of Gamborg’s B5 medium

| **Metabolite** | **Formula** | **Mol wt.(g/mol)** | **Conc. (mg/L)**  **(Defined)** | **mmol/L in media**  **(Defined)** | **Conc. (mg/L)**  **0^th^ day** | **Conc. (mg/L)**  **15^th^ day** | **Uptake (mg/L)** | **mmol/L** | **Flux (mmol/g DW/h)** |
| --- | --- | --- | --- | --- | --- | --- | --- | --- | --- |
| Sucrose | C12H22O11 | 342.30 | 30000 | 87.64 | 20338.38 | 586.904 | 19751.47 | 57.70 | **0.0142** |
| Ammonium | NH4+ | 18.04 | 36.59 | 2.03 | 26.24 | 2.88 | 23.36 | 1.29 | **0.0003** |
| Nitrate | NO3- | 62.01 | 1533.21 | 24.73 | 1226.50 | 907.08 | 319.42 | 5.15 | **0.0013** |
| Potassium | K+ | 39.10 | 966.96 | 24.73 | 1156.00 | 695.33 | 460.67 | 11.78 | **0.0029** |
| Phosphate | H2PO4- | 96.99 | 105.43 | 1.09 | - | - | - | 1.09 | **0.0003** |
| Sulphate | SO42- | 96.07 | 210.83 | 2.19 | - | - | - | 2.19 | 0.0005 |
| Calcium | Ca2+ | 40.08 | 40.93 | 1.02 | 44.01 | 16.92 | 27.09 | 0.68 | **0.0002** |
| Chloride | Cl- | 70.90 | 72.40 | 1.02 | - | - | - | 1.02 | 0.0003 |
| Magnesium | Mg+2 | 24.31 | 24.65 | 1.01 | 22.62 | 0.82 | 21.80 | 0.90 | **0.0002** |
| Sodium | Na+ | 22.99 | 24.99 | 1.09 | 50.55 | 40.19 | 10.36 | 0.45 | 0.0001 |
| Boric acid | H3BO3 | 61.84 | 3.00 | 0.05 | - | - | - | 0.05 | 0.0000 |
| Cobalt | Co2+ | 58.93 | 0.01 | 0.00 | - | - | - | 0.00 | 0.0000 |
| Copper | Cu2+ | 63.55 | 0.01 | 0.00 | - | - | - | 0.00 | 0.0000 |
| EDTA | C10H16N2O8 | 372.24 | 37.30 | 0.10 | - | - | - | 0.10 | 0.0000 |
| Ferrous | Fe2+ | 55.84 | 5.58 | 0.10 | 7.22 | 5.91 | 1.31 | 0.02 | 0.0000 |
| Manganese | Mn2+ | 54.94 | 3.25 | 0.06 | - | - | - | 0.06 | 0.0000 |
| Molybdic acid | H2MoO4 | 190.00 | 0.21 | 0.00 | - | - | - | 0.00 | 0.0000 |
| Iodide | I- | 126.90 | 0.57 | 0.00 | - | - | - | 0.00 | 0.0000 |
| Zinc | Zn2+ | 65.40 | 0.45 | 0.01 | - | - | - | 0.01 | 0.0000 |
| myo-Inositol | C6H12O6 | 180.16 | 100.00 | 0.56 | - | - | - | 0.56 | 0.0001 |
| Nicotinic acid | C6H5NO2 | 123.11 | 1.00 | 0.01 | - | - | - | 0.01 | 0.0000 |
| Pyridoxine HCl | C8H12ClNO3 | 205.64 | 1.00 | 0.00 | - | - | - | 0.00 | 0.0000 |
| Thiamine HCl | C12H18Cl2N4OS | 337.30 | 10.00 | 0.03 | - | - | - | 0.03 | 0.0000 |
| Water | H2O | 18.02 | 14.57 | 0.81 | - | - | - | 0.81 | 0.0002 |

Table 2b. Media uptake constraints incorporated into the model

**Biomass Reaction**

Based on the above data, the formation of 1 g of dry cell mass of *N. nimmoniana* (including growth-associated maintenance energy requirements, GAM) is defined by the following equation:

30 H2O[c0] + 0.0003 NAD[c0] + 30.027 ATP[c0] + 0.00013 NADP[c0] + 0.00015 NADH[c0] + 0.0001 NADPH[c0] + 0.000136 CoA[c0] + 0.027 UTP[c0] + 0.0001 FAD[c0] + 0.0001 FMN[c0] + 0.0001 Pyridoxal_phosphate[c0] + 0.000543 S_Adenosyl_methionine[c0] + 0.043 L_Methionine[c0] + 0.037 L_Malate[c0] + 0.114 L_Glutamate[c0] + 0.067 L_Glutamine[c0] + 0.117 L_Alanine[c0] + 0.026 GTP[c0] + 0.0757 Oxaloacetate[c0] + 0.013 Citrate[c0] + 0.092 L_Aspartate[c0] + 0.026 CTP[c0] + 0.080 cpd00132[c0] + 0.163 L_Serine[c0] + 0.0001 TPP[c0] + 0.118 Glycine[c0] + 0.076 L_Phenylalanine[c0] + 0.039 L_Lactate[c0] + 0.049 L_Tyrosine[c0] + 0.087 L_Threonine[c0] + 0.13 Glycerol_3_phosphate[c0] + 0.034 L_Cysteine[c0] + 0.0001 Tetrahydrofolate[c0] + 0.0001 10_Formyltetrahrofolate[c0] + 0.0001 5_10_Methylenetetrahydrofolate[c0] + 0.0001 5_Methyltetrahydrofolate[c0] + 0.03 dATP[c0] + 0.0001 5_10_Methenyltetrahydrofolate[c0] + 0.088 L_Proline[c0] + 0.086 cis_Aconitate[c0] + 0.766 UDP_xylose[c0] + 0.55 4_Coumarate[c0] + 0.026 dGTP[c0] + 0.03 TTP[c0] + 0.076 Ferulate [c0] + 0.0001 5_Formyltetrahydrofolate[c0] + 0.026 dCTP[c0] + 0.001 Phosphopantetheine[c0] + 0.307 K_plus[c0] + 0.023 L_Tryptophan[c0] + 0.114 L_Valine[c0] + 0.094 L_Arginine[c0] + 0.21 Cl[c0] + 0.329 Palmitate[c0] + 0.043 L_Histidine[c0] + 0.097 L_Isoleucine[c0] + 0.173 L_Leucine[c0] + 0.105 L_Lysine[c0] + 0.011 Stearic_Acid[c0] + 1.59 Alpha_D_Glucose[c0] + 0.2271 Beta_D_Fructose[c0] + 0.0377 Beta_D_Ribofuranose[c0] + 0.0001 Biotin[c0] + 0.000975 Phytonadiol[d0] + 0.015 Oleate[c0] + 0.0001 ubiquinol9[c0] + 0.000155 Plastoquinol_9[c0] + 0.0066 Ascorbate[c0] + 0.0026 Beta_carotene[c0] + 0.00033 Vitamin_E[c0] -> 30 Pi[c0] + 0.218 PPi[c0] + 30 H_plus[c0] + 30 ADP[c0] + 0.766 UDP[c0] + 1 g Biomass

The suffix ‘[c0]’ is applied to metabolites’ abbreviation to indicate their occurrence in the cytoplasm.

**Examples of gap-filling and manual curation**

During manual curation of NothaGEM, the reaction of glycerate oxidoreductase involves conversion of glycerate to hydroxypyruvate in the plastid. However, hydroxypyruvate was detected as dead-end. Hydroxypyruvate is known to produce L-serine (PMN database). Hence, a corresponding reaction involving serine biosynthesis has been added to the model.

**
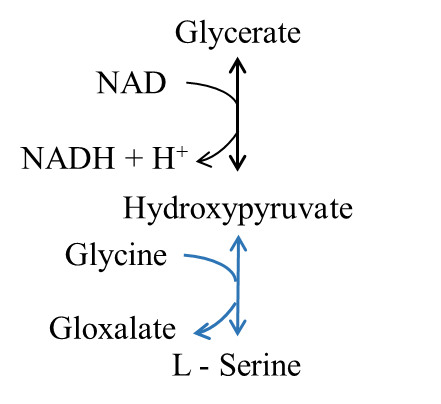
**

In the glyoxalate shunt, the reaction involving conversion of threo-isocitrate to succinate and glyoxalate is well-known. However, isocitrate was detected as a dead-end. Reaction involving isocitrate glyoxalate lyase was added to the model.

**
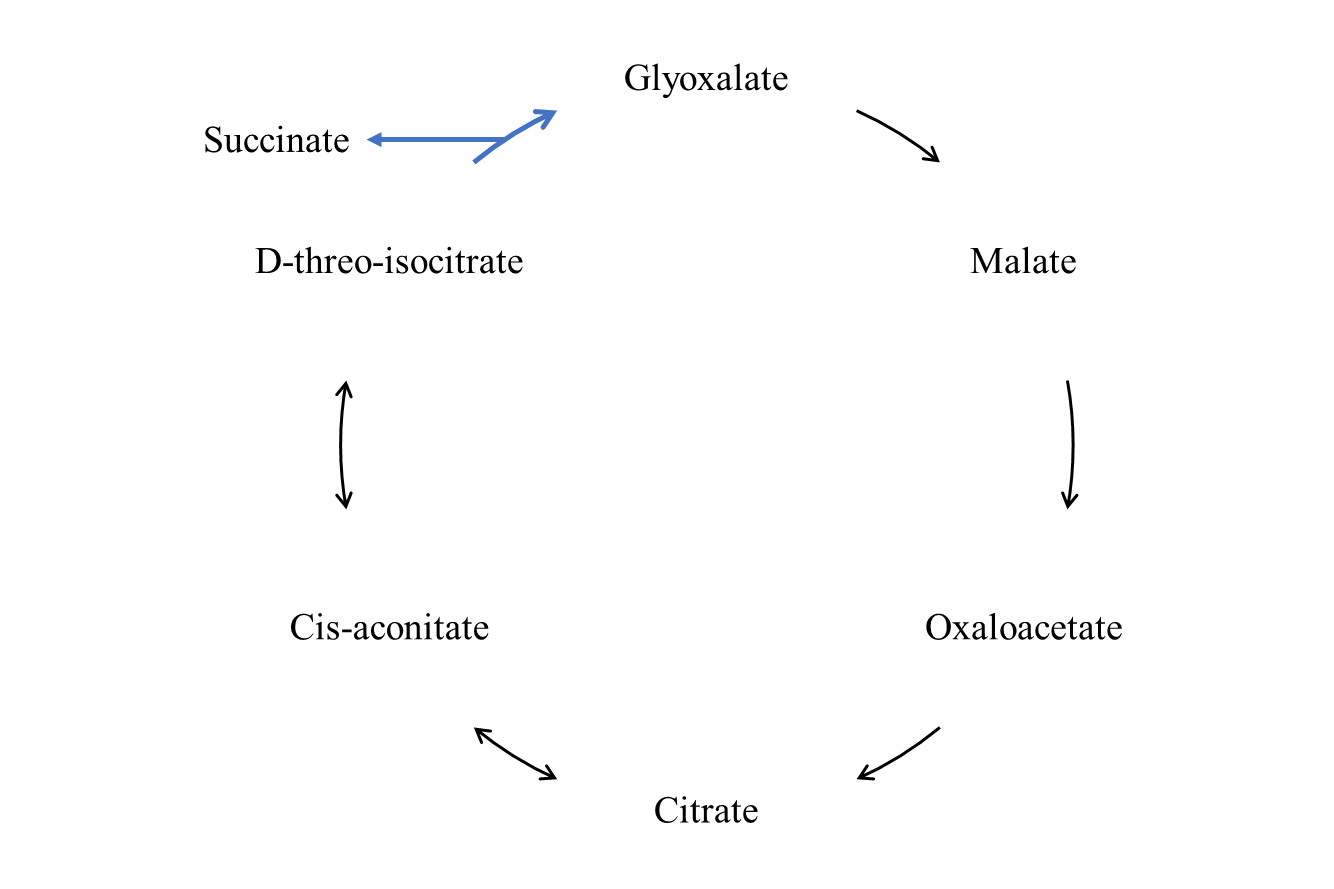
**

Fig. 5 The standard curve of Camptothecin obtained by plotting various concentrations of camptothecin against their respective peak area from HPLC

Table 3. List of oligonucleotide primers used for amplification

| # | Cell line | Camptothecin yield (µg/g) |
| --- | --- | --- |
| 1 | STR 2 | 4.77 ± 0.3 |
| 2 | STR 8 | 4.37 ± 0.2 |
| 3 | WN 1 | 0.87 ± 0.1 |

Table 4. Camptothecin yield from callus cultures of *N. nimmoniana.* STR – strictosidine synthase callus, WN – Wildtype *N. nimmoniana* callus

**References**

- Bligh, E. G., & Dyer, W. J. (1959). A rapid method of total lipid extraction and purification. *Canadian journal of biochemistry and physiology*, *37*(8), 911-917. <https://doi.org/10.1139/o59-099>
- Cataldo, D.A., Haroon, M.H., Schrader, L.E., Youngs, V.L., 2008. Rapid colorimetric determination of nitrate in plant tissue by nitration of salicylic acid. <https://doi.org/10.1080/00103627509366547> 6, 71–80.
- Feist, A.M., Palsson, B.O., 2010. The biomass objective function. Curr. Opin. Microbiol. 13, 344–349. https://doi.org/10.1016/J.MIB.2010.03.003
- Lingwan, M., & Masakapalli S. K. (2022). Deciphering the metabolic adjustments of engineered plants using GC-MS: A typical workflow. *The Future of Metabolic Engineering*, 197-213. <https://doi.org/10.52305/VEAH4499>
- Lingwan, M., Shagun, S., Pahwa, F., Kumar, A., Verma, D. K., Pant, Y., ... & Masakapalli, S. K. (2021). Phytochemical rich Himalayan Rhododendron arboreum petals inhibit SARS-CoV-2 infection in vitro. *Journal of Biomolecular Structure and Dynamics*, 1-11. <https://doi.org/10.1080/07391102.2021.2021287>
- Lisec, J., Schauer, N., Kopka, J., Willmitzer, L., & Fernie, A. R. (2006). Gas chromatography mass spectrometry–based metabolite profiling in plants. *Nature protocols*, *1*(1), 387-396. <https://doi.org/10.1038/nprot.2006.59>
- Lommen, A., & Kools, H. J. (2012). MetAlign 3.0: performance enhancement by efficient use of advances in computer hardware. *Metabolomics*, *8*(4), 719-726. <https://doi.org/10.1007/s11306-011-0369-1>.
- Martínez‐García, J. F., Monte, E., & Quail, P. H. (1999). A simple, rapid and quantitative method for preparing Arabidopsis protein extracts for immunoblot analysis. *The Plant Journal*, *20*(2), 251-257. <https://doi.org/10.1046/j.1365-313x.1999.00579.x>
- Masakapalli, S. K., Bryant, F. M., Kruger, N. J., & Ratcliffe, R. G. (2014). The metabolic flux phenotype of heterotrophic Arabidopsis cells reveals a flexible balance between the cytosolic and plastidic contributions to carbohydrate oxidation in response to phosphate limitation. *The Plant Journal*, *78*(6), 964-977. <https://doi.org/10.1111/tpj.12522>
- Rather, G.A., Sharma, A., Pandith, S.A., Kaul, V., Nandi, U., Misra, P., Lattoo, S.K., 2018. De novo transcriptome analyses reveals putative pathway genes involved in biosynthesis and regulation of camptothecin in Nothapodytes nimmoniana (Graham) Mabb. Plant Mol. Biol. 96, 197–215. https://doi.org/10.1007/S11103-017-0690-9/FIGURES/7
- Solorzano, L., 1969. Determination of ammonia in natural waters by the phenol hypochlorite method. Deep Sea Res. 18, 531–532.
- Thiele, I., Palsson, B., 2010. A protocol for generating a high-quality genome-scale metabolic reconstruction. Nat. Protoc. 5, 93. https://doi.org/10.1038/NPROT.2009.203
