# Supplementary material for: Genome-scale metabolic model led engineering of Nothapodytes nimmoniana plant cells for high camptothecin production
Source: Front Plant Sci. 2023 Aug 2;14:1207218. doi: 10.3389/fpls.2023.1207218 (PMC10433906; doi:10.3389/fpls.2023.1207218)

M_cam Expand All Readme 2022-04-24 12:28

Independent Section

Contains tests that are independent of the class of modeled organism, a model's [illegible]

Consistency

Stoichiometric Consistency	100.0%
Mass Balance	92.0%
Charge Balance	95.0%
Metabolite Connectivity	100.0%
Unbounded Flux In Default Medium	50.0%
Sub Total	91%

Annotation - Metabolites

Presence of Metabolite Annotation	100.0%
Metabolite Annotations Per Database	Info
pubchem.compound	0.0%
kegg.compound	92.3%
seed.compound	100.0%
inchikey	0.0%
inchi	0.0%
chebi	0.0%
hmdb	0.0%
reactome	0.0%
metanetx.chemical	0.0%
bigg.metabolite	0.0%
biocyc	0.0%
Metabolite Annotation Conformity Per Database	Info
pubchem.compound	0.0%
kegg.compound	100.0%
seed.compound	100.0%
inchikey	0.0%
inchi	0.0%
chebi	0.0%

[illegible]

Specific Section

Covers general statistics and specific aspects of a metabolic network that are not [illegible]

SBML

SBML Level and Version	Errored
FBC enabled	Errored

Basic Information

Model Identifier	M_cam
Total Metabolites	1,228
Total Reactions	1,233
Total Genes	1,809
Total Compartments	11
Metabolic Coverage	0.68
Uncoserved Metabolites	0
Minimal Inconsistent Net Stoichiometries	Skipped

Metabolite Information

Unique Metabolites	1,228
Duplicate Metabolites in Identical Compartments	0
Metabolites without Charge	0
Metabolites without Formula	0
Medium Components	23

Reaction Information

Purely Metabolic Reactions	914
Purely Metabolic Reactions with Constraints	0
Transport Reactions	294
Transport Reactions with Constraints	2
Reactions With Partially Identical Annotations	0.34
Duplicate Reactions	0.00

[illegible]

M_cam Expand All Readme 2022-04-24 12:28

metanetx.chemical	0.0%	
bigg.metabolite	0.0%	
biocyc	0.0%	
Uniform Metabolite Identifier Namespace	0.0%	
Sub Total	34%	

Annotation - Reactions

Presence of Reaction Annotation	100.0%
Reaction Annotations Per Database	Info
rhea	0.0%
kegg.reaction	59.2%
seed.reaction	95.0%
metanetx.reaction	0.0%
bigg.reaction	0.0%
reactome	0.0%
ec-code	0.0%
brenda	0.0%
biocyc	0.0%
Reaction Annotation Conformity Per Database	Info
rhea	0.0%
kegg.reaction	100.0%
seed.reaction	100.0%
metanetx.reaction	0.0%
bigg.reaction	0.0%
reactome	0.0%
ec-code	0.0%
brenda	0.0%
biocyc	0.0%
Uniform Reaction Identifier Namespace	99.5%
Sub Total	60%

Annotation - Genes

Presence of Gene Annotation	0.0%

Gene-Protein-Reaction (GPR) Associations

Reactions without GPR	142
Fraction of Transport Reactions without GPR	0.12
Enzyme Complexes	0

Biomass

Biomass Reactions Identified	1
Biomass Consistency	1.01
Biomass Production In Default Medium	0.00
Unrealistic Growth Rate In Default Medium	false
Biomass Production In Complete Medium	206.81
Blocked Biomass Precursors In Default Medium	0
Blocked Biomass Precursors In Complete Medium	0
Ratio of Direct Metabolites in Biomass Reaction	0.19
Number of Missing Essential Biomass Precursors	37

Energy Metabolism

Non-Growth Associated Maintenance Reaction	Errored
Growth-associated Maintenance in Biomass Reaction	false
Number of Reversible Oxygen-Containing Reactions	9
Erroneous Energy-generating Cycles	Info
MNXM3	Skipped
MNXM63	Skipped
MNXM51	Skipped
MNXM121	Skipped
MNXM423	Skipped

M_cam Expand All Readme 2022-04-24 12:28

uniprot	0.0%	
ecogene	0.0%	
kegg.genes	0.0%	
ncbigi	0.0%	
ncbigene	0.0%	
ncbiprotein	0.0%	
ccds	0.0%	
hprd	0.0%	
asap	0.0%	
Gene Annotation Conformity Per Database	Info	
refseq	0.0%	
uniprot	0.0%	
ecogene	0.0%	
kegg.genes	0.0%	
ncbigi	0.0%	
ncbigene	0.0%	
ncbiprotein	0.0%	
ccds	0.0%	
hprd	0.0%	
asap	0.0%	
Sub Total	0%	

Annotation - SBO Terms

Metabolite General SBO Presence	100.0%
Metabolite SBO:0000247 Presence	100.0%
Reaction General SBO Presence	100.0%
Metabolic Reaction SBO:0000176 Presence	95.4%
Transport Reaction SBO:0000185 Presence	78.6%
Exchange Reaction SBO:0000627 Presence	100.0%
Demand Reaction SBO:0000628 Presence	Skipped
Sink Reactions SBO:0000632 Presence	Skipped
Gene General SBO Presence	0.0%

MNXM38	Skipped
MNXM208	Skipped
MNXM191	Skipped
MNXM223	Skipped
MNXM7517	Skipped
MNXM12233	Skipped
MNXM558	Skipped
MNXM21	Skipped
MNXM89557	Skipped

Network Topology

Universally Blocked Reactions	321
Orphan Metabolites	86
Dead-end Metabolites	96
Stoichiometrically Balanced Cycles	397
Metabolite Production In Complete Medium	448
Metabolite Consumption In Complete Medium	469

Matrix Conditioning

Ratio Min/Max Non-Zero Coefficients	0.00
Independent Conservation Relations	195
Rank	1033
Degrees Of Freedom	200

Experimental Data Comparison

Growth Prediction	Skipped
Gene Essentiality Prediction	Skipped

Misc. Tests

Environment

Python Version	3.8.12

M_cam Expand All Readme 2022-04-24 12:28

SBO:0000629 Presence [illegible]

Sub Total 61%

Total Score 67%

Total Score

67%

Score per Category

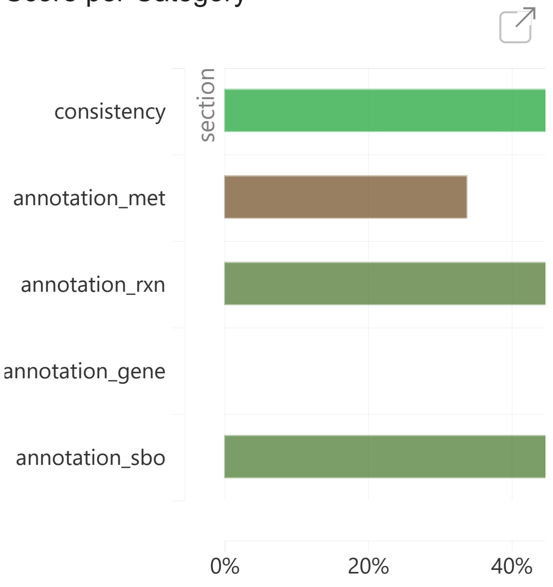

Supplement: Supplementary Data Sheet 2 — NothaGEM model parameters, comprising - S2.1 – Metabolites, S2.2 – Reactions, S2.3 –Memote report, S2.4 – Model (SBML file). [file DataSheet_2.zip › Dataset S2/Memote Report Dataset S2.3.pdf]
